# Circular RNAs and complex diseases: from experimental results to computational models

**DOI:** 10.1093/bib/bbab286

**Published:** 2021-07-30

**Authors:** Chun-Chun Wang, Chen-Di Han, Qi Zhao, Xing Chen

**Affiliations:** School of Information and Control Engineering, China University of Mining and Technology; School of Information and Control Engineering, China University of Mining and Technology; School of Computer Science and Software Engineering, University of Science and Technology Liaoning; China University of Mining and Technology

**Keywords:** circRNA, disease, circRNA-disease association prediction, network algorithm, machine learning, computational model

## Abstract

Circular RNAs (circRNAs) are a class of single-stranded, covalently closed RNA molecules with a variety of biological functions. Studies have shown that circRNAs are involved in a variety of biological processes and play an important role in the development of various complex diseases, so the identification of circRNA-disease associations would contribute to the diagnosis and treatment of diseases. In this review, we summarize the discovery, classifications and functions of circRNAs and introduce four important diseases associated with circRNAs. Then, we list some significant and publicly accessible databases containing comprehensive annotation resources of circRNAs and experimentally validated circRNA-disease associations. Next, we introduce some state-of-the-art computational models for predicting novel circRNA-disease associations and divide them into two categories, namely network algorithm-based and machine learning-based models. Subsequently, several evaluation methods of prediction performance of these computational models are summarized. Finally, we analyze the advantages and disadvantages of different types of computational models and provide some suggestions to promote the development of circRNA-disease association identification from the perspective of the construction of new computational models and the accumulation of circRNA-related data.

## CircRNA

Circular RNAs (circRNAs) are a class of single-stranded, covalently closed RNA molecules, which are produced by backsplicing from pre-mRNAs [1]. During backsplicing, a downstream splice-acceptor site is covalently connected to an upstream splice-donor site [1]. The first circRNA molecules, viroids, were identified more than 40 years ago [2, 3]. Soon after, Hsu *et al.* [4] discovered circRNAs in the cytoplasmic fractions of eukaryotic cell lines through electron microscopy. Furthermore, circRNAs were identified to be produced from self-splicing introns of pre-ribosomal RNA in unicellular eukaryotes [5]. Later, researcher discovered that a small part of circRNAs stem from protein-coding genes in archaea [6]. However, circRNAs were initially treated as ‘junk’ yielded by splicing errors [7].

As the development of high-throughput RNA sequencing technology and new bioinformatics algorithms, more and more circRNAs were discovered in eukaryotes including protists, fungi, plants, insects and mammals [8–13]. CircRNAs are a relatively large family of RNAs and massive circRNAs have been identified, but studies on the classification of circRNAs and the mechanism of loop formation have just begun. CircRNAs mainly include exonic circRNAs (ecircRNAs), exon-intron circRNAs (EIciRNAs) and circular intronic RNAs (ciRNAs) [14]. Among them, ecircRNAs are produced by the exons in the back-splicing process of pre-mRNA, which are abundant in the cytoplasm [15]. The EIciRNAs are widely present in the nucleus, which are formed by the combined action of exons and introns during the back-splicing process [16]. In addition, ciRNAs are formed by introns and are mainly localized in the nucleus [17]. Besides, circRNAs could be generated from more than 10% of expressed gene in the investigated cells and tissues [18, 19]. It can be learned that the expression of circRNAs is broad. Usually, the expression level of circRNA is low [20, 21], but some circRNAs are experimentally verified to be high expressed in specific type of cells or tissues [15, 22]. Moreover, thousands of circRNAs are abundant in the mammalian brain and some of them are upregulated during neurogenesis [23]. These studies demonstrate that circRNAs should not be‘junk’ and they may have specifically biological functions.

### CircRNA function

CircRNAs are usually expressed in only a few cell types, exhibiting significant specificity during tissue and developmental stages. However, some other circRNAs show cross-species conservation [18]. In addition, by comparison with linear exons, the exon sequence of circRNA appears to be more conserved at the third codon position, while the third codon is meaningless at the protein level [21]. These indicate that in addition to encoding proteins, circRNA has other functions.

#### CircRNAs as microRNA sponges

In 2013, Hansen *et al*. [24] found that hsa_circRNA_105055 has more than 70 miR-7 binding sites. Further functional studies have showed that ciRS-7 strongly restrains the activity of miR-7, which in turn leads to an increase in the target level of miR-7. They also demonstrated that hsa_circRNA_105055 and miR-7 have overlapping co-expression in mouse brain tissue [24]. In addition, the sex-determining region Y (Sry)^9^ of hsa_circRNA_105055 has 16 microRNA (miRNA)-138 binding sites [24]. Moreover, researchers have demonstrated that circ-HIPK3, circ-ITCH and mm9-circ-012559 can act as miRNA sponges [25–27]. The above findings indicate that circRNA is very common as miRNA sponge.

#### CircRNAs regulate the expression of parental genes

Different types of circRNAs have different ways of regulating their parental genes. Specifically, ciRNAs promote transcription of genes by binding to Pol II. Zhang *et al*. [17] found that knocking out ciRNA can suppress the expression of its parental gene. For the specific ciRNA ci-ankrd52, it aggregates into the transcriptional site and acts as a positive regulator of Pol II transcription. For EIciRNA, it binds to U1 snRNP to form EIciRNA-U1 snRNP complexes, which further binds to Pol II, thereby promoting transcription of the parental gene [17]. Besides, Li *et al*. [16] found that EIciRNAs can regulate gene expression in the nucleus, which mainly enhances the expression of the parental gene in *cis* and affects transcriptional regulation through the interaction between U1 snRNA and EIciRNA. In addition, ecircRNA, containing miRNA response elements, can bind to miRNA and indirectly regulate the expression of its parent mRNA. Li *et al*. [28] found that hsa_circRNA_001141 binds to miR-7 and miR-214 in lung cancer cells and enhances the expression of ITCH, thereby inhibiting the activity of Wnt/β-catenin.

#### Competition with pre-mRNA splicing

The pre-mRNA can undergo typical linear splicing to produce mRNA during processing, while nonlinear splicing generates circRNA. Recent studies have found that increasing the efficiency of linear splicing can significantly reduce the abundance of circRNA [29]. When the length of the introns flanking the circRNA is longer, the efficiency of typical linear splicing is reduced, while the efficiency of cyclization is increased [30]. The above findings indicate that circRNA can compete with the pre-mRNA during transcription.

### CircRNA-disease associations

Previous functional analysis of circRNAs has demonstrated that a circRNA, hsa_circRNA_105055, contains more than 70 miRNA target sites and can act as a miRNA sponge [24]. Besides, some studies have indicated that circRNAs can regulate protein functions [16, 31]. As biological functions of circRNAs were discovered, circRNAs are receiving the attention of researchers. In the field of human health, more and more studies have shown that circRNAs have close associations with human complex diseases [32–34]. In the following, we will introduce several common cancers and their associated circRNAs.

#### Gastric cancer

Gastric cancer, one of the top five cancers in the world [35]. In 2019, 27 510 patients were newly diagnosed with gastric cancer and 11 140 patients died because of gastric cancer in the USA [36]. Therefore, it is necessary to discover and explore pathogenesis for the early diagnosis, prevention and treatment of gastric cancer. So far, increasing experiments have shown that circRNAs play an irreplaceable role in the development of gastric cancer [37]. Li *et al*. [38] found that there were 343 differentially expressed (DE) circRNAs by comparing the gastric cancer patients’ plasma and plasma of healthy control, and then, the two techniques of reverse-transcription real-time polymerase chain reaction (RT-PCR) [39] and RT-droplet digital PCR (RT-ddPCR) [40] were used to determine the expression level of circRNAs. More concretely, patients with low expression levels of hsa_circ_0001017 or hsa_circ_0061276 in plasma have a shorter overall survival than patients with higher expression levels [38]. In addition, circRNA-0026 regulates RNA transcription, RNA metabolism and gene expression in gastric cancer [41]. Moreover, biological studies have found that knocking out hsa_circ_0047905, hsa_circ_0138960 and has-circRNA7690–15 in gastric cancer cells down-regulates the expression of the parental gene [42]. Inhibition of the expression of these three circRNAs can inhibit the proliferation and invasion of gastric cancer cells [42].

#### Breast cancer

Breast cancer is one of the major cancer types among women worldwide, and 12% of women are diagnosed with breast cancer during their lifetime in the USA [43]. Common symptoms of breast cancer include: a lump in the breast, a change in breast shape and red or scaly skin. CircRNAs are closely related to the formation and development of breast cancer, and recent studies have found that the expression of some circRNAs can be used to prevent breast cancer [44–46]. For example, hsa_circ_0001982 in breast cancer tissues inhibits breast cancer cell proliferation and induces apoptosis by targeting miR-143 [44]. In addition, knocking out hsa_circRNA_005239 can inhibit the proliferation and promote the apoptosis in triple negative breast cancer [46]. There are also some circRNAs that can be used as potential biomarkers for breast cancer detection. For example, Yin *et al.* [45] found that the expression level of hsa_circ_0001785 in plasma of breast cancer patients is significantly different from that in preoperative, postoperative and healthy individuals, which demonstrates that hsa_circ_0001785 can act as a diagnostic biomarker for breast cancer.

#### Lung cancer

Lung cancer is characterized by uncontrolled growth of cells in the lung tissue. It is reported that 85% of lung cancer is caused by long-term smoking [47]. Other factors that cause lung cancer include genetic factors, secondhand smoke or air pollution [48, 49]. The circRNA of hsa_circRNA_001141 in lung cancer tissues has been shown to suppress the development of lung cancer by enhancing the expression of its parental gene ITCH [28], while hsa_circ_0013958 in lung cancer cells can promote the proliferation of lung cancer cells and inhibit apoptosis [50]. Besides, Yao *et al.* [51] found that circRNA_100876 is abnormally expressed in non-small cell lung cancer. In addition, the higher the expression level of circRNA_100876, the lower the survival rate [51]. Therefore, circRNA_100876 can be used as biomarker for early detection and screening of lung cancer.

#### Pancreatic cancer

Pancreatic cancer is usually caused by uncontrolled growth, division and spread of cells in the pancreas [52]. Symptoms usually manifest as digestive problems including: weight loss, indigestion, back pain, nausea and so on [53]. Studies have found that smoking or lack of exercise and long-term heavy drinking may lead to chronic pancreatitis [54]. Guo *et al*. [55] demonstrated the dysregulation of circRNA expression in pancreatic cancer tissues using qRT-PCR. In addition, they predicted that multiple circRNAs have complementary sequences to miR-15a / miR-506 and different miRNA binding sites in the seed region [55]. Furthermore, Chen *et al*. [56] found that circRNA_100782 regulates the proliferation of BxPC3 pancreatic cancer cells by interacting with miR-124.

There is increasing evidence that circRNAs are related with the development and invasion of complex diseases, although most of the action mechanisms are still unknown [57]. Besides, circRNAs could be novel biomarkers for human cancers [58]. Therefore, identifying associations between circRNAs and diseases would facilitate the diagnosis, prevention and prognosis of human complex diseases.

## Databases

Data collection about circRNAs, diseases and circRNA-disease associations is an important premise when researchers identify novel circRNA-disease associations by bioinformatics methods. In addition, the systematic collection and management of the information about circRNAs and circRNA-disease relationships is important for further inspection of the underlying molecular mechanism of circRNAs. In this section, we introduce some important databases, from which researchers could obtain circRNA related data more conveniently. These databases can be divided into two categories. Specifically, the first type of databases record circRNA-disease associations (see Table 1). The second type of databases provide comprehensive annotation resources for circRNAs (see Table 2). More detailed introduction of these databases can be seen from Supplementary Materials available online at https://academic.oup.com/bib.

**Table 1:** Databases recording circRNA-disease associations

Database	Number of circRNAs	Number of diseases	Number of associations	URL
Circ2Traits [59]	1951	105	Unknown	http://gyanxet-beta.com/circdb/
Circ2Disease [60]	237	54	273	http://bioinformatics.zju.edu.cn/Circ2Disease/index.html
CircR2Disease [61]	661	100	725	http://bioinfo.snnu.edu.cn/CircR2Disease/
CircRNADisease [33]	330	48	354	http://cgga.org.cn:9091/circRNADisease/
Circad [62]	1338	720	1338	http://clingen.igib.res.in/circad/

**Table 2:** Databases providing annotation resources for circRNAs

Database	Number of circRNAs	Short description	URL
circBase [63]	92 375	Provides information of circRNAs including the genomic position, gene symbols, evidence for the occurrence	http://www.circbase.org/
CircNet [64]	34 000	Provides the information of circRNA expression profiles, circRNA-miRNA sponge regulatory network, circRNA-gene-miRNA regulatory network	http://circnet.mbc.nctu.edu.tw/
deepBase v2.0 [65]	14 867	Provides comprehensive expression and evolution profiles of circRNAs.	http://biocenter.sysu. edu.cn/deepBase/
circRNADb [66]	32 914	Provides the information of protein-coding potential of circRNAs	http://reprod.njmu.edu.cn/circrnadb
TSCD [67]	302 853	Provides the genomic location and conservation of tissue specific circRNAs	http://gb.whu.edu.cn/TSCD
CSCD [68]	272 152	Records the function and regulation of cancer-associated circRNAs	http://gb.whu.edu.cn/CSCD
CIRCpedia v2 [69]	262 782	Records the information of location, strand, isoform, expression value, sequencing type and conservation of circRNAs	https://www.picb.ac.cn/rnomics/circpedia/
exoRBase [70]	58 330	Provides circRNA expression profile, expression rank, gene symbol and spliced length	http://www.exorbase.org/
CircFunBase [71]	7059	Provides the information of circRNA function, GO annotations and circRNA-associated miRNAs	http://bis.zju.edu.cn/CircFunBase/
TRCirc [72]	92 375	Contains more than 765 000 transcription factor-circRNA relationships	http://www.licpathway. net/TRCirc
circbank [73]	140 790	Develops a new naming system based on the host genes of circRNAs	http://www.circbank.cn/
CircRiC [74]	92 589	Provides the modules of integrative analysis, drug response, biogenesis, and expression landscape	https://hanlab.uth.edu/cRic/
MiOncoCirc [75]	227 056	Records circRNAs from metastases, primary tumors, and very rare cancer types	https://nguyenjoshvo.github.io/
VirusCircBase [76]	11 924	Provides the information of the location, genes involved in the viral circRNA, the abundance, the detection method	http://www.computational biology.cn/ViruscircBase/home.html

## Computational models

As the development of high-throughput sequencing technology and bioinformatics analysis methods, more and more circRNAs are identified. However, the function and mechanism of circRNAs are unclear in most cases. In addition, researchers discover that the occurrence and development of various diseases including cancer are associated with circRNAs. Identifying and studying circRNA-disease associations is important for understanding the function and molecular mechanism of circRNAs. In addition, circRNA-disease association identification is meaningful for the early detection, early diagnosis and effective treatment of diseases. However, it is time-consuming and laborious to discover novel circRNA-disease relationships directly by biological experiments. Computational models could effectively predict potential circRNA-disease associations for further experimental verification, which would save many resources.

During recent years, scientists have successively proposed some computational models for predicting potential circRNA-disease associations based on distinct algorithms. These computational models can be roughly divided into two categories, namely network algorithm-based models and machine learning-based models (see Table 3). In this section, we mainly introduce the general steps of construction of different models and the main advantages or limitations of these models. The main symbols utilized throughout this sections are listed in Table 4.

**Table 3:** List of different types of circRNA-disease prediction models

Model name	Core algorithm	Model type	Source code
PCWCDA	DFS algorithm	Network algorithm-based model	Unavailable
BRWSP	Biased random walk algorithm	Network algorithm-based model	Unavailable
KATZHCDA	KATZ	Network algorithm-based model	Unavailable
KATZCPDA	KATZ	Network algorithm-based model	Unavailable
IBNPKATZ	Bipartite network projection algorithm and KATZ	Network algorithm-based model	Unavailable
NCPCDA	Network consistency projection	Network algorithm-based model	Unavailable
DWNCPCDA	DeepWalk and network consistency projection	Network algorithm-based model	Unavailable
LLCDC	LLC and label propagation algorithm	Network algorithm-based model	Unavailable
CD-LNLP	Label propagation algorithm	Network algorithm-based model	Unavailable
DWNN-RLS	Regularized least squares of kronecker product kernel	The first type of machine learning-based model	Unavailable
RWRLCDA	Random work and logistic regression	The first type of machine learning-based model	Unavailable
MRLDC	Manifold regularization-learning	The first type of machine learning-based model	Unavailable
iCircDA-MF	Matrix factorization	The first type of machine learning-based model	Unavailable
GMCDA	Graph-based multi-label learning	The first type of machine learning-based model	Unavailable
iCDA-CMG	Collective Matrix completion	The first type of machine learning-based model	Unavailable
NMFIBAC	Non-negative matrix factorization	The first type of machine learning-based model	Unavailable
SIMCCDA	Speedup inductive matrix completion	The first type of machine learning-based model	https://github.com/bioinformaticsAHU /SIMCCDA
PreCDA	PersonalRank algorithm	The first type of machine learning-based model	https://github.com/wythit/PreCDA
ICFCDA	Collaboration filtering	The first type of machine learning-based model	Unavailable
RWRKNN	Random walk with restart and KNN	The second type of machine learning-based model	Unavailable
iCDA-CGR	SVM	The second type of machine learning-based model	Unavailable
GBDTCDA	GBDT	The second type of machine learning-based model	Unavailable
DFPUCDA	DF	The second type of machine learning-based model	https://github.com/xzenglab/DeepDCR
CNNCDA	CNN	The second type of machine learning-based model	Unavailable
GCNCDA	Graph Convolutional Network	The second type of machine learning-based model	Unavailable
AE-DNN	Autoencoder and DNN	The second type of machine learning-based model	Unavailable
AE-RF	Autoencoder and RF	The second type of machine learning-based model	https://github.com/Deepthi-K523 /AE-RF

**Table 4:** The main symbols utilized throughout the Computational models section

Symbol	Definition and description
A^*^	Adjacency matrix of heterogeneous network
${W}_{cg}$	Weight matrix of circRNA graph
${W}_{dg}$	Weight matrix of disease graph
${L}_c$	Laplacian matrix of circRNA graph
${L}_d$	Laplacian matrix of disease graph
*CS*	CircRNA Similarity matrix
*CSS*	CircRNA Semantic Similarity matrix
*CFS*	CircRNA Functional Similarity matrix
*CES*	CircRNA Expression Similarity matrix
*CTS*	CircRNA Topological Similarity matrix
*RCS*	Reconstructed CircRNA Similarity matrix
*DS*	Disease Similarity matrix
*DSS*	Disease Semantic Similarity matrix
*DTS*	Disease Topological Similarity matrix
*RDS*	Reconstructed Disease Similarity matrix
*KC*	GIP Kernel similarity matrix of CircRNA
*KD*	GIP Kernel similarity matrix of Disease
*GS*	Gene Similarity matrix
*CD*	CircRNA-Disease association matrix
*CG*	CircRNA-Gene interaction matrix
*GD*	Gene-Disease association matrix
*AS*	The predicted circRNA-disease Association Score matrix
*c_i_*	CircRNA *i*
*d_j_*	Disease *j*
${N}_c$	The number of circRNAs
${N}_d$	The number of diseases
*N*(*c_i_*)	The neighbors of *c_i_*
*N*(*d_j_*)	The neighbors of *d_j_*

### Network algorithm-based models

In network algorithm-based models, circRNA similarity network, disease similarity network and circRNA-disease association network are usually utilized to construct a heterogeneous network. Then, the corresponding algorithm is used to predict potential relationships based on the heterogeneous network.

#### PWCDA

**Figure 1:**
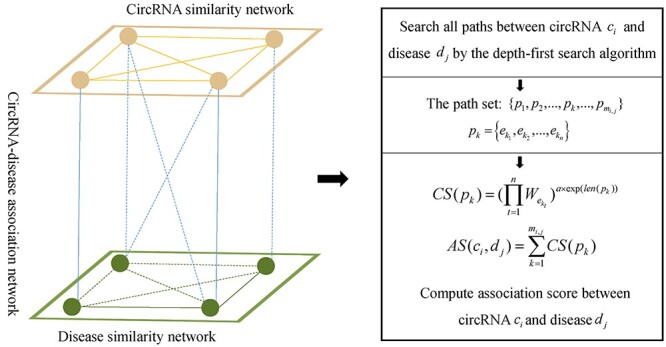
The workflow of PWCDA to infer potential circRNA-disease associations based on DFS algorithm to search paths on a heterogeneous network.

Lei *et al.* [77] developed the model of Path Weighed method for predicting CircRNA-Disease Associations (PWCDA) (see Figure 1). The same model has been used for potential miRNA-disease association prediction before [78]. They first construct a heterogeneous network, which is composed of circRNA similarity network, disease similarity network and circRNA-disease association network. Then, PWCDA searches all the paths between circRNA ${c}_i$ and disease ${d}_j$ with the length less than $\eta$ by depth-first search (DFS) algorithm. The path set can be described as $\{{p}_1,{p}_2,\dots, {p}_k,\dots, {p}_{m_{i,j}}\}$, where the variable ${m}_{i,j}$ denotes the number of searched paths between circRNA ${c}_i$ and disease ${d}_j$. Finally, the predicted score between ${c}_i$ and ${d}_j$ can be calculated by accumulating all contributing scores (*CS*) of paths in $\{{p}_1,{p}_2,\dots, {p}_k,\dots, {p}_{m_{i,j}}\}$. The $CS({p}_k)$ of the path ${p}_k=\{{e}_{k_1},{e}_{k_2},\dots, {e}_{k_n}\}$ is defined as follows:

(1) \begin{equation*} CS\left({p}_k\right)={\left(\prod \limits_{t=1}^n{W}_{e_{k_t}}\right)}^{a\times \exp \left(\mathrm{len}\left({p}_k\right)\right)} \end{equation*}

where ${W}_{e_{k_t}}$ is the weight of the edge ${e}_{k_t}$ in the path ${p}_k$. Besides, $\alpha$ is a constraint factor and $\mathrm{len}({p}_k)$ denotes the length of ${p}_k$. The decaying function $\alpha \times \exp (\mathrm{len}({p}_k))$ is used to further reduce the CS of long paths. Then, the final association score between ${c}_i$ and ${d}_j$ is defined as follows:

(2) \begin{equation*} AS\left({c}_i,{d}_j\right)=\sum \limits_{k=1}^{m_{i,j}} CS\left({p}_k\right) \end{equation*}

In PCWDA, only paths within three steps are used to decrease the noisy information. However, the decaying function in PCWDA is relatively simple.

#### BRWSP

**Figure 2:**
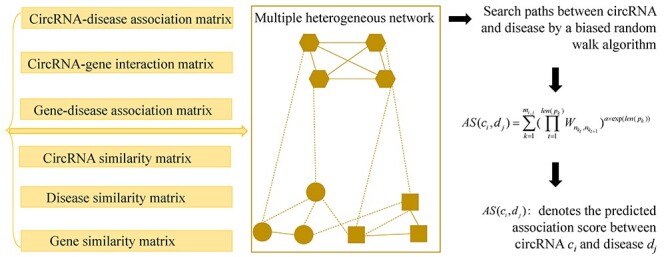
The flowchart of BRWSP to predict circRNA-disease associations based on biased random walk to search paths on a multiple heterogeneous network.

Lei *et al.* [79] proposed a computational model (see Figure 2) of Biased Random Walk to Search Paths on a multiple heterogeneous network (BRWSP) to predict circRNA-disease associations. Firstly, they construct the multi-layer heterogeneous network by utilizing the information of circRNA similarity matrix *CS*, disease similarity matrix *DS*, gene similarity matrix *GS*, circRNA-disease association matrix *CD*, circRNA-gene interaction matrix *CG* as well as gene-disease association matrix *GD*. The heterogeneous network is represented as follows:

(3) \begin{equation*} {A}^{\ast }=\left(\begin{array}{ccc} CS& CG& CD\\{}{CG}^T& GS& GD\\{}{CD}^T& {GD}^T& DS\end{array}\right) \end{equation*}

To avoid the biases caused by larger values in ${A}^{\ast }$, a normalized multi-layer heterogeneous network denoted by $\mathrm{NMH}={D}^{-(1/2)}{A}^{\ast }{D}^{-(1/2)}$ is further established, where *D* is the degree matrix of ${A}^{\ast }$.

Secondly, a biased random walk algorithm is employed to search paths between circRNAs and diseases in the heterogeneous network. Specifically, the random walker starts from the investigated circRNA node *u* and first randomly moves to one neighbor of *u*. Then, the walker continues to walk to the next node. Here, ${c}_k$ is employed to denote the node accessed by the walker on its *k*th move. The strategy of selecting the next node is described as follows:

(4) \begin{equation*} \begin{array}{l}P\left({c}_{k+1}=x|{c}_k=v,{c}_{k-1}=t\right)=\\{}\kern0.1em \left\{\begin{array}{@{}l}\displaystyle\frac{\Phi \left(t,v,x\right)\ast \mathrm{NMH}\left(v,x\right)}{\sum_{i\in \mathrm{Nei}(v)}\mathrm{NMH}\left(v,i\right)},\mathrm{if}\ x\in \mathrm{Nei}(v)\kern0.2em \mathrm{and}\kern0.2em x\notin \left\{{c}_0,{c}_1,\dots, {c}_k\right\}\\{}0,\kern9em \mathrm{otherwise}\end{array}\right.\end{array} \end{equation*}

(5) \begin{equation*} \Phi \left(t,v,x\right)=\left\{\begin{array}{@{}l}q,\kern1.75em \mathrm{if}\ x\in \mathrm{Nei}(v)\ \mathrm{and}\ x\in \mathrm{Nei}(t)\\{}1-q,\kern0.5em \mathrm{otherwise}\end{array}\right.\kern-4pt \end{equation*}

where $P({c}_{k+1}=x|{c}_k=v,{c}_{k-1}=t)$ represents the transition probability from the current node *v* to the next node *x* when the last visited node is the node *t*. Besides, $\mathrm{Nei}(v)$ and $\mathrm{Nei}(t)$ denote the neighbors of the current node *v* and the last visited node *t* in the heterogeneous network, respectively. For the parameter *q*, if *q* is assigned a larger value, the biased random walk algorithm tends to select the nodes near the investigated node. Otherwise, the biased random walk algorithm tends to select the nodes away from the investigated node. It can be seen from Eq. (4) that the next accessed node will be chosen from the neighbors of the current nodes based on their probability. The random walker keeps moving until the investigated disease node is accessed. ${p}_k=\{{n}_{k_1},{n}_{k_2},\dots{n}_{k_i},\dots, {n}_{k_{L+1}}\}$ is used to denote one path between circRNA ${n}_{k_1}$ and disease ${n}_{k_{L+1}}$, where ${n}_{k_i}$ represents the node (circRNA, disease or gene) of ${p}_k$ and *L* is the length of ${p}_k$. To search more paths between investigated circRNA and disease, the above process will be repeated. Only paths with lengths less than *L* will be left. The set $\{{p}_1,{p}_2,\dots, {p}_{m_{i,j}}\}$ is utilized to denote the searched paths, where ${m}_{i,j}$ is the number of paths between circRNA ${c}_i$ and disease ${d}_j$.

Finally, the association score $AS({c}_i,{d}_j)$ between circRNA ${c}_i$ and disease ${d}_j$ can be computed as follows:

(6) \begin{equation*} AS\left({c}_i,{d}_j\right)=\sum \limits_{k=1}^{m_{i,j}}{\left(\prod \limits_{t=1}^{\mathrm{len}\left({p}_k\right)}{W}_{n_{k_t},{n}_{k_{t+1}}}\right)}^{a\times \exp \left(\mathrm{len}\left({p}_k\right)\right)} \end{equation*}

where ${W}_{n_{k_t},{n}_{k_{t+1}}}$ denotes the weight of the edge connecting the node ${n}_{k_t}$ and ${n}_{k_{t+1}}$. In addition, $\alpha$ is a decay factor and $\mathrm{len}({p}_k)$ is the length of ${p}_k$.

#### KATZHCDA

**Figure 3:**
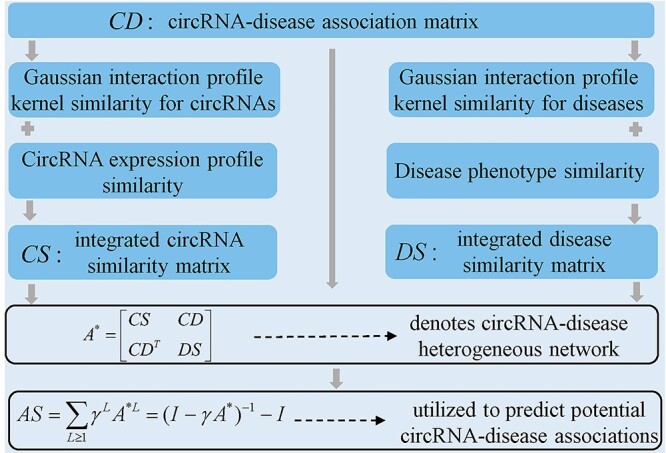
The flow diagram of KATZHCDA to predict human circRNA-disease associations based on KATZ algorithm.

Fan *et al.* [80] established a calculation model (see Figure 3) of KATZ-based Human CircRNA-Disease Association prediction (KATZHCDA). KATZ measure is a network-based method, which computes similarity of nodes in a heterogeneous network to solve the problem of association prediction [81, 22]. In KATZHCDA, the authors first compute the integrated similarity for circRNAs and diseases, which are denoted by the matrices of *CS* and *DS*, respectively. Besides, the association matrix *CD* is employed to denote the information of circRNA-disease associations, and $CD(i,j)$ is equal to 1 if circRNA ${c}_i$ is associated with disease ${d}_j$, otherwise 0. Secondly, circRNA similarity network, disease similarity network as well as circRNA-disease association network are combined to construct a heterogeneous network whose adjacency matrix can be described as follows:

(7) \begin{equation*} {A}^{\ast }=\left[\begin{array}{l} CS\kern1.45em CD\\{}{CD}^T\kern0.75em DS\end{array}\right] \end{equation*}

The number of walks between circRNA nodes and disease nodes, as well as the length of walks are two key similarity metrics in the heterogeneous network. Because the contribution of longer walks is lower than that of shorter walks, the parameter $\gamma$ is utilized to control the contribution of walks with different lengths. The final association score between ${c}_i$ and ${d}_j$ can be defined as follows:

(8) \begin{equation*} AS\left({c}_i,{d}_j\right)=\sum \limits_{L=1}^K{\gamma}^L{A}^{\ast L}\left(i,j\right) \end{equation*}

where the variable *L* denotes the length of walk and the variable *K* is the user specified parameter. Equation (8) can be transformed into the matrix form

(9) \begin{equation*} AS=\sum \limits_{L\ge 1}{\gamma}^L{A}^{\ast L}={\left(I-\gamma{A}^{\ast}\right)}^{-1}-I \end{equation*}

where *AS* can be used to predict potential circRNA-disease associations. As walks with longer length may be insignificant, the variable *K* is normally set as 2, 3 and 4, respectively. One advantage of KATZHCDA lies that it can predict circRNA-disease association scores for all diseases simultaneously. Besides, KATZHCDA can predict associated circRNAs for new diseases without any known associations.

#### KATZCPDA

Deng *et al*. [83] developed the model of KATZCPDA based on the KATZ method and the information of circRNA, protein and disease. Because the number of circRNA-disease associations validated by experiments is insufficient, they first obtain inferred circRNA-disease relationships by utilizing protein-circRNA association network and protein-disease association network based on the principle of gilt-by-association, that is biological objects are more likely to be associated if they have the same or related behavior [84]. Then, they construct a heterogeneous network by integrating the circRNA similarity network denoted by matrix *CS*, the disease similarity network denoted by matrix *DS* and the circRNA-disease association network denoted by *CD*, which combines the experimentally confirmed circRNA-disease associations and inferred circRNA-disease associations. The heterogeneous network can be represented as follows:

(10) \begin{equation*} {A}^{\ast }=\left[\begin{array}{l} CS\kern1.6em CD\\{}{CD}^T\kern0.8000001em DS\end{array}\right] \end{equation*}

Next, the final circRNA-disease association matrix is obtained in the similar way as KATZHCDA. KATZCPDA introduces the bridge of protein to obtain inferred circRNA-disease relationships, which increases the number of associations and the quantity of heterogeneous network.

#### IBNPKATZ

**Figure 4:**
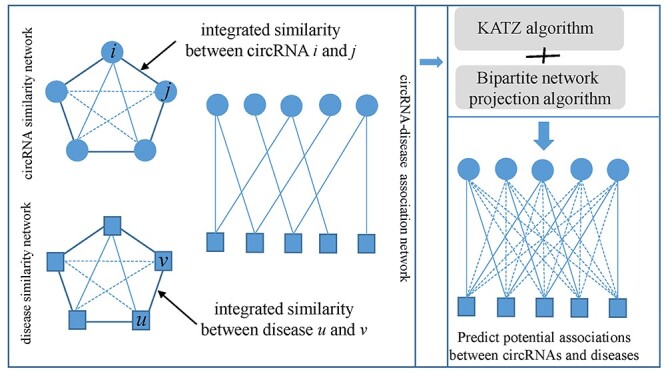
The workflow of IBNPKATZ to infer circRNA-disease associations based on bipartite network projection algorithm and KATZ algorithm.

Zhao *et al*. [85] raised a novel circRNA-disease association prediction model (see Figure 4) by Integrating Bipartite Network Projection algorithm and KATZ measure (IBNPKATZ). Firstly, in the bipartite network projection algorithm, resource scores of circRNAs are used to be the association scores for a given disease. Specifically, a hierarchical clustering algorithm is utilized to construct circRNAs’ bias ratings which denote the association degree between diseases and their associated circRNAs from circRNAs’ perspective. For disease ${d}_i$, the bias rating of its related circRNA ${c}_j$ can be computed as follows:

(11) \begin{equation*} r\left({d}_i,{c}_j\right)=\frac{n_{\mathrm{cr}}\left({c}_j\right)}{T\left({d}_i\right)} \end{equation*}

where ${n}_{\mathrm{cr}}({c}_j)$ is the number of circRNAs in the cluster cr including ${c}_j$ and $T({d}_i)$ denotes the number of circRNAs related with ${d}_i$. For ${d}_i$, the initial resource score of its related circRNA ${c}_j$ can be calculated by normalizing the bias rating of ${c}_j$ as follows:

(12) \begin{equation*} \hat{r}\left({d}_i,{c}_j\right)=\frac{r\left({d}_i,{c}_i\right)T\left({d}_i\right)}{\sum_{k=1}^{N_c}r\left({d}_i,{c}_k\right)} \end{equation*}

where ${N}_c$ is the number of circRNAs. Then, circRNAs associated with ${d}_i$ allocate their resource score to their associated diseases as follows:

(13) \begin{equation*} {R}_{cd}\left({d}_i,{c}_j\right)=\frac{\hat{r}\left({d}_i,{c}_j\right)}{\sum_{k=1}^{N_d}\hat{r}\left({d}_k,{c}_j\right)}\times \hat{r}\left({d}_i,{c}_j\right) \end{equation*}

where ${N}_d$ is the number of diseases. Next, the diseases distribute their received resource score to their associated circRNAs as follows:

(14) \begin{equation*} {R}_{dc}\left({d}_i,{c}_j\right)=\frac{r\left({d}_i,{c}_j\right)}{\sum_{k=1}^{N_c}r\left({d}_i,{c}_k\right)}\times{\sum}_{k=1}^{N_c}{R}_{cd}\left({d}_i,{c}_k\right) \end{equation*}

The final resource score of cricRNA ${c}_j$ for given disease ${d}_i$ can be computed as follows:

(15) \begin{equation*} {R}_{\mathrm{fin}}\left({c}_j|{d}_i\right)=\sum \limits_{k=1}^{N_d}{R}_{dc}\left({d}_k,{m}_j\right) \end{equation*}

Similarly, the final resource score ${R}_{\mathrm{fin}}({d}_i|{c}_j)$ of disease ${d}_i$ for circRNA ${c}_j$ could be obtained. Finally, the predicted circRNA-disease association score based on the bipartite network projection algorithm is defined as

(16) \begin{equation*} {S}_{\mathrm{BNP}}\left({d}_i,{c}_j\right)=\frac{R_{\mathrm{fin}}\left({c}_j|{d}_j\right)+{R}_{\mathrm{fin}}\left({d}_i|{c}_j\right)}{2} \end{equation*}

Secondly, the authors utilize KATZ measure on the heterogeneous network, constructed by using information of integrated circRNA similarity, integrated disease similarity and known circRNA-disease relationships, to predict circRNA-disease association score ${S}_{\mathrm{KATZ}}({d}_i,{c}_j)$ in the similar way as KATZHCDA. Finally, the circRNA-disease association scores of ${S}_{\mathrm{BNP}}({d}_i,{c}_j)$ and ${S}_{\mathrm{KATZ}}({d}_i,{c}_j)$ are integrated as the final association score

(17) \begin{equation*} AS\left({d}_i,{c}_j\right)=\frac{S_{\mathrm{BNP}}\left({d}_i,{c}_j\right)+{S}_{\mathrm{KATZ}}\left({d}_i,{c}_j\right)}{2} \end{equation*}

Combination of two different prediction algorithms contributes to the ideal predictive performance of IBNPKATA.

#### NCPCDA

**Figure 5:**
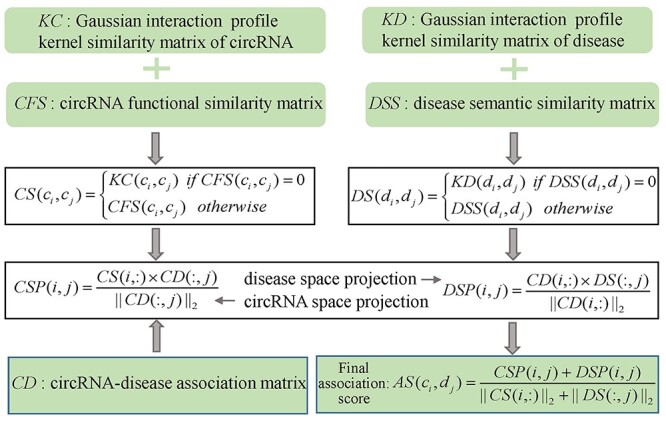
The flowchart of NCPCDA for circRNA-disease association prediction based on the circRNA space projection and disease space projection.

Li *et al*. [86] raised a calculation model (see Figure 5) of Network Consistency Projection for inferring CircRNA-Disease Association (NCPCDA). In NCPCDA, the binary matrix *CD* denotes the circRNA-disease associations. Besides, $CS$ and $DS$ represent integrated similarity matrices of circRNAs and diseases, respectively. The circRNA similarity and disease similarity are defined as follow:

(18) \begin{equation*} CS\left({c}_i,{c}_j\right)=\left\{\begin{array}{@{}l} KC\left({c}_i,{c}_j\right)\kern0.7em \mathrm{if}\kern0.2em CFS\left({c}_i,{c}_j\right)=0\\{} CFS\left({c}_i,{c}_j\right)\kern1em \mathrm{otherwise}\end{array}\right. \end{equation*}

(19) \begin{equation*} DS\left({d}_i,{d}_j\right)=\left\{\begin{array}{@{}l} KD\left({d}_i,{d}_j\right)\kern0.7em \mathrm{if}\kern0.2em DSS\left({d}_i,{d}_j\right)=0\\{} DSS\left({d}_i,{d}_j\right)\kern1em \mathrm{otherwise}\end{array}\right.\kern-0pt \end{equation*}

where $KC$ and $KD$ denote the Gaussian interaction profile (GIP) kernel similarity matrices of circRNAs and diseases, respectively. Besides, the matrices $CFS$ and $DSS$ are circRNA functional similarity matrix and disease semantic similarity matrix, respectively. NCPCDA is made up of circRNA space projection $CSP$ and disease space projection $DSP$, which are defined as

(20) \begin{equation*} CSP\left(i,j\right)=\frac{CS\left(i,:\right)\times CD\left(:,j\right)}{{\left\Vert CD\left(:,j\right)\right\Vert}_2} \end{equation*}

(21) \begin{equation*} DS P\left(i,j\right)=\frac{CD\left(i,:\right)\times DS\left(:,j\right)}{{\left\Vert CD\left(i,:\right)\right\Vert}_2} \end{equation*}

where $CS(i,:)$ and $CD(i,:)$ are the *i*th rows of $CS$ and $CD$, respectively. Besides, $DS(:,j)$ and $CD(:,j)$ are the *j*th columns of $DS$ and $CD$, respectively. In the end, the final associations score $AS({c}_i,{d}_j)$ between circRNA ${c}_i$ and disease ${d}_j$ can be calculated by integrating and normalizing $CSP$ and $DSP$ as follows:

(22) \begin{equation*} AS\left({c}_i,{d}_j\right)=\frac{CSP\left(i,j\right)+ DSP\left(i,j\right)}{{\left\Vert CS\left(i,:\right)\right\Vert}_2+{\left\Vert DS(:,j)\right\Vert}_2} \end{equation*}

No parameters appear in NCPCDA, which reduces the complexity of prediction process. However, the similarity of circRNA is calculated only based on known circRNA-disease associations, which leads to the failure of NCPCDA for predicting associated diseases for cirRNAs without any known related diseases.

#### DWNCPCDA

Li *et al*. [87] developed the DeepWalk and Network Consistency Projection-based algorithm to predict CircRNA-Disease Association (DWNCPCDA). In most of circRNA-disease association prediction models, the circRNA similarity and disease similarity are usually calculated by multiple biological information of circRNAs and diseases. In this study, the authors construct circRNA topological similarity matrix *CTS* and disease topological similarity matrix *DTS* only based on circRNA-disease association network. More formally, the DeepWalk algorithm [88] is utilized to learn circRNA representations stored by the matrix $CR$ and disease representations stored by the matrix $DR$ based on the circRNA-disease association network. DeepWalk obtains local information of input graph by truncated random walk and utilizes them to learn latent representations of vertices in the input graph [88]. Then, similarity between circRNAs or diseases can be computed as follows:

(23) \begin{equation*} CTS\left({c}_i,{c}_j\right)=\frac{\sum \limits_{k=1}^d CR\left({c}_i,k\right)\times CR\left({c}_j,k\right)}{\sqrt{\sum \limits_{k=1}^d CR{\left({c}_i,k\right)}^2}\sqrt{\sum \limits_{k=1}^d CR{\left({c}_j,k\right)}^2}} \end{equation*}

(24) \begin{equation*} DTS\left({d}_i,{d}_j\right)=\frac{\sum \limits_{k=1}^d DR\left({d}_i,k\right)\times DR\left({d}_j,k\right)}{\sqrt{\sum \limits_{k=1}^d DR{\left({d}_i,k\right)}^2}\sqrt{\sum \limits_{k=1}^d DR{\left({d}_j,k\right)}^2}} \end{equation*}

where the variable *d* is the dimension of representations of circRNAs and diseases.

After obtaining *CTS* and *DTS*, network consistency projection method, which have been used in the prediction model of NCPCDA, is adopt to calculate circRNA-disease association matrix *AS*. Although similarity of circRNA and disease is computed only based on the circRNA-disease association network, DWNCPCDA still achieves good predictive accuracy, which demonstrates the excellent ability of DeepWalk in learning latent representations of circRNAs and diseases.

#### L‌LCDC

**Figure 6:**
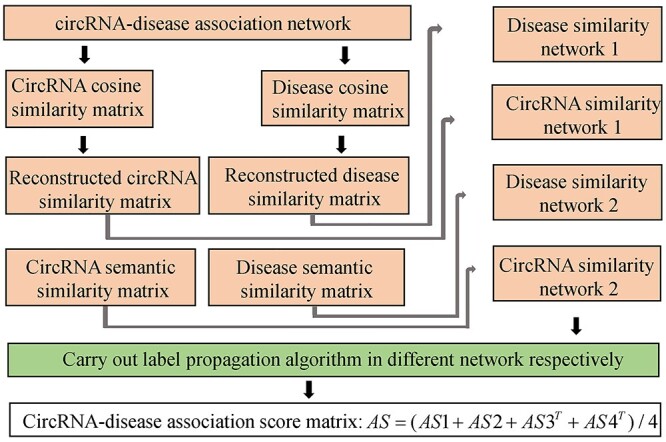
The framework of LLCDC to predict potential circRNA-disease associations based on LLC and label propagation algorithm.

Ge *et al*. [89] proposed a computational model of LLCDC (see Figure 6) to predict potential circRNA-disease associations based on locality-constrained linear coding (LLC) and label propagation algorithm. Firstly, they calculate circRNA semantic similarity matrix $CSS$ based on GO terms of circRNA-related genes. Besides, disease semantic matrix $DSS$ is calculated based on MeSH descriptors of diseases. Secondly, they also calculate cosine similarity matrices of circRNAs and diseases based on circRNA-disease association information and further utilized LLC to obtain reconstructed circRNA similarity matrix $RCS$ and reconstructed disease similarity matrix $RDS$ based on above two cosine similarity matrices. Thirdly, label propagation algorithm is employed to obtain the initial predicted circRNA-disease association matrix *AS*1 based on circRNA semantic similarity network by the following iterative equation:

(25) \begin{equation*} AS1\left(t+1\right)=\theta \times CSS\times AS1(t)+\left(1-\theta \right) CD \end{equation*}

where $AS1(0)= CD$ and $\theta$ are used to control the utilization of similarity and association information. $AS1(t)$ denotes the association matrix obtained in the *t*th iteration. The iterative equation will be conducted until *AS*1 converges. In a similar way, label propagation algorithm is carried out based on $DSS$, $RCS$ and $RDS$ to obtain association matrices $AS2$, $AS3$ and $AS4$, which are combined as the finally predicted association matrix *AS* as follows:

(26) \begin{equation*} AS=\frac{1}{4}\left( AS1+ AS2+ AS{3}^T+ AS{4}^T\right) \end{equation*}

#### CD-LNLP

Zhang *et al*. [90] put forward a computational method to infer CircRNA-Disease associations based on a Linear Neighborhood similarity measure and Label Propagation algorithm (CD-LNLP). The information of associations between ${N}_c$ circRNAs and ${N}_d$ diseases is recorded in the binary matrix *CD*. In CD-LNLP, linear neighborhood similarity (LNS) measure is utilized to construct circRNA similarity matrix *CS* and disease similarity matrix *DS*. In LNS, the *i*th row vector of *CD* is considered as the feature profile of circRNA ${c}_i$. The basic idea of LNS is that each feature profile of circRNA can be reconstructed by the linear combination of feature profiles of neighbors of the circRNA, which can be formulated as follows:

(27) \begin{equation*} {\displaystyle \begin{array}{l}\underset{CS}{\min}\kern0.3em \frac{1}{2}{\left\Vert CD-\left(C\ast CS\right) CD\right\Vert}_F^2+\frac{\mu }{2}\sum \limits_{i=1}^{N_c}{\left\Vert{\left(C\ast CS\right)}_{i.}\right\Vert}_1^2\\{}s.t.\kern0.8000001em \left(C\ast CS\right)e=e,\kern1em CS\ge 0\end{array}} \end{equation*}

where $\ast$ is the Hadamard product. The matrix *C* with the size of ${N}_c\times{N}_c$ is an indicator matrix, whose element $C(i,j)$ is equal to 1 if circRNA ${c}_j$ is one of the *K* nearest neighbors (by Euclidean distance) of circRNA ${c}_i$; otherwise, $C(i,j)=0$. Besides, ${(C\ast CS)}_{i.}$ is the *i*th row of $C\ast CS$. In addition, *e* is a ${N}_c\times 1$ vector and all elements in *e* are 1. The first item of above formula is the loss function of LNS. The second item is used to achieve row sparsity of $C\ast CS$. The constraint condition is used to ensure that the sum of similarity values between any circRNA and its neighbors is equal to 1. By utilizing Lagrange multiplier method to solve the optimization problem, they obtain the update rule for *CS*

(28) \begin{equation*} CS\left(i,j\right)=\left\{\begin{array}{@{}c} CS\left(i,j\right)\frac{{\left({\boldsymbol{CDCD}}^T+{ee}^T\right)}_{i,j}}{{\left(\left(C\ast CS\right){\boldsymbol{CDCD}}^T+\mu \left(C\ast CS\right){ee}^T\right)}_{ij}}i\ne j\\{}0\kern0.5em i=j\end{array}\right.\kern-4pt \end{equation*}

In a similar way, disease similarity matrix *DS* can be obtained. Next, a label process [91] is employed to predicted potential circRNA-disease relationships, which can be formulated as follows:

(29) \begin{equation*} {AS}_{\mathrm{cicRNA}}=\left(1-\alpha \right){\left(I-\alpha CS\right)}^{-1} CD \end{equation*}

(30) \begin{equation*} {AS}_{\mathrm{disease}}=\left(1-\alpha \right){\left(I-\alpha DS\right)}^{-1}{CD}^T \end{equation*}

where the ${N}_c\times{N}_d$ matrix ${AS}_{\mathrm{circRNA}}$ and the ${N}_d\times{N}_c$ matrix ${AS}_{\mathrm{disease}}$ are the predicted association matrix based on circRNA similarity and disease similarity, respectively. Finally, the integrated association scores between circRNAs and diseases can be computed as follows:

(31) \begin{equation*} AS=\rho{AS}_{\mathrm{circRNA}}+\left(1-\rho \right){AS}_{\mathrm{disease}}^T \end{equation*}

where the parameter $\rho$ is utilized to regulate the weight of ${AS}_{\mathrm{circRNA}}$ and ${AS}_{\mathrm{disease}}$. The application of LNS measure contributes to the effectiveness of CD-LNLP. However, the similarity of diseases and circRNAs is calculated only based on circRNA-disease association network.

### Machine learning-based models

Machine learning algorithms have been successfully used in many fields of association prediction [92–101]. In the last few years, researchers utilized different machine learning methods to construct prediction models for the identification of potential circRNA-disease associations. These machine learning-based models can be further roughly divided into two types. The first type of models can obtain the predictive association matrix by directly solving specific optimization problem, such as regularized least squares, manifold regularization learning, matrix decomposition and inductive matrix completion algorithm-based models. In addition, the second type of models train classifier to infer circRNA-disease association, such as logistic regression-, K-Nearest Neighbor (KNN)-, Support Vector Machines (SVM)-, Random Forest (RF)-, Gradient Boosting Decision Tree (GBDT)-, Deep Forest (DF)-, Convolutional Neural Network (CNN)-, Graph Neural Network (GNN)- and Deep Neural Network (DNN)-based models. When feature vector of a sample is input into classifier, the classifier can output an association score for the sample. Furthermore, some prediction models combine different algorithms to improve the prediction accuracy.

### The first type of machine learning-based models

#### DWNN-RLS

**Figure 7:**
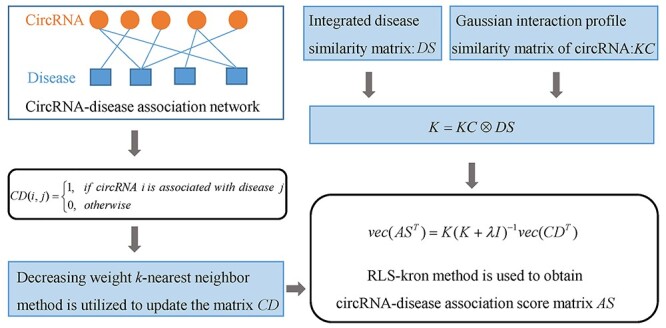
The workflow of DWNN-RLS to infer potential circRNA-disease associations based on regularized least squares of kronecker product kernel.

Yan *et al*. [102] developed a computational model, called as DWNN-RLS (see Figure 7) to infer potential circRNA-disease associations based on regularized least squares of kronecker product kernel (RLS-kron). In DWNN-RLS, the matrix $CD$ is utilized to denote the information of known circRNA-disease relationships. In addition, the disease similarity matrix $DS$ is obtained by integrating disease GIP kernel similarity matrix *KD* and disease semantic similarity matrix *DSS*. In this study, the authors first utilize DWNN (decreasing weight KNN) method to calculate the initial association score between new circRNA ${c}_i$ and disease $d{}_j$ as follows:

(32) \begin{equation*} {AS}_{\mathrm{initial}}\left({c}_i,{d}_j\right)=\frac{\sum \limits_{c_l\in N\left({c}_i\right)} KC\left({c}_i,{c}_l\right)\times CD\left({c}_l,{d}_j\right)}{\sum \limits_{c_l\in N\left({c}_i\right)} KC\left({c}_i,{c}_l\right)} \end{equation*}

where the new circRNA ${c}_i$ means that ${c}_i$ has no known associated disease. In addition, $N({c}_i)$ is the set of all neighbors of ${c}_i$. Similarly, the initial association score between new disease $d{}_j$ and circRNA ${c}_i$ can be calculated as follows:

(33) \begin{equation*} {AS}_{\mathrm{initial}}\left({c}_i,{d}_j\right)=\frac{\sum _{d_l\in N\left({d}_j\right)} KD\left({d}_i,{d}_l\right)\times CD\left({c}_i,{d}_l\right)}{\sum _{d_l\in N\left({d}_j\right)} KD\left({d}_i,{d}_l\right)} \end{equation*}

where $N({d}_j)$ represents the set of all neighbors of ${d}_i$. Then, they employ the RLS-kron method to infer new associations between circRNAs and diseases as follows:

(34) \begin{equation*} \mathrm{vec}\left({AS}^T\right)=K{\left(K+\lambda I\right)}^{-1}\mathrm{vec}\left({CD}^T\right) \end{equation*}

where the kernel $K= KC\otimes DS$ is the Kronecker product of $KC$ and $DS$. As $KC$ and $DS$ are real symmetric matrices, the two matrices can be decomposed as follows:

(35) \begin{equation*} KC={\vee}_c\,{\wedge}_c\,{\vee}_c^T \end{equation*}

(36) \begin{equation*} DS={\vee}_d{\,\wedge}_d{\,\vee}_d^T \end{equation*}

where the columns of the matrices of ${\vee}_c$ and ${\vee}_d$ are the eigenvectors of $KC$ and $DS$, respectively. Besides, ${\wedge}_c$ and ${\wedge}_d$ are diagonal matrices whose diagonal elements are the eigenvalues of $KC$ and $DS$, respectively. Thus, the finally predicted circRNA-disease association matrix can be computed as follows:

(37) \begin{equation*} AS={\vee}_c{Z}^T{\,\vee}_d^T \end{equation*}

(38) \begin{equation*} \mathrm{vec}(Z)=\left({\wedge}_c\otimes{\wedge}_d\right){\left({\wedge}_c\otimes{\wedge}_d+\lambda I\right)}^{-1}\mathrm{vec}\left({\vee}_d^T{CD}^T{\,\vee}_c\right) \end{equation*}

#### RWLRCDA

Ding *et al.* [103] built a computational model based on Random Walk and Logistic Regression to infer CircRNA-Disease Associations (RWLRCDA). Specifically, they first calculate the circRNA similarity matrix *CS* and construct circRNA similarity network where vertex ${c}_i$ and ${c}_j$ are connected by an edge with the weight value of $CS({c}_i,{c}_j)$. Subsequently, aiming to obtain the global relationship information of each circRNA, the authors treat each circRNA as seed node in turn and utilize the random walk with restart algorithm on circRNA similarity network to obtain related circRNAs for the seed node with corresponding probability. Next, they extract three features, namely *pos*, *neg* and *label*, for each pair of circRNA ${c}_i$ and disease ${d}_j$. Specifically, ${C}_k(i)$ denotes the set of top-*k* circRNAs related with ${c}_i$. The *pos* value is the sum of probability of circRNAs which are in ${C}_k(i)$ and related with ${d}_j$. Similarly, The *neg* value is the sum of probability of circRNAs which are in ${C}_k(i)$ and not related with ${d}_j$. The *label* value is 1 or 0. Finally, logistic regression is utilized to predict the association score for circRNA-disease pair as follows:

(39) \begin{equation*} AS(x)=\frac{\exp \left(w\times x\right)}{1+\exp \left(w\times x\right)} \end{equation*}

where *x* is the feature vector consisting of three features (*pos*, *neg* and *label*) of the circRNA-disease pair and *w* is the weight vector which can be trained by maximizing the posterior association probability of circRNA-disease training samples as follows:

(40) \begin{equation*} w=\arg \kern0.2em \max \left(\prod_{i=1}^mp\left({y}_i|{x}_i\right)\right) \end{equation*}

(41) \begin{equation*} p\left({y}_i=1|{x}_i\right)=\frac{\exp \left(w\times{x}_i\right)}{1+\exp \left(w\times{x}_i\right)} \end{equation*}

(42) \begin{equation*} p\left({y}_i=0|{x}_i\right)=\frac{1}{1+\exp \left(w\times{x}_i\right)} \end{equation*}

where *m* is the number of training samples. Besides, ${x}_i$ and ${y}_i$ are the feature vector and label of the *i*th circRNA-disease sample. RWLRCDA can predict associations for new diseases or new circRNAs. However, RWLRCDA utilizes too little information of diseases.

#### MRLDC

**Figure 8:**
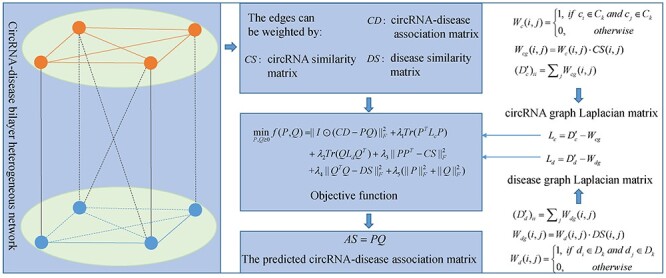
The flowchart of MRLDC for predicting human disease-associated circRNAs based on a manifold regularization-learning framework.

Xiao *et al*. [103] developed a manifold regularization-learning framework, called MRLDC, for predicting human disease-associated circRNAs (see Figure 8). They construct a circRNA-disease bilayer heterogeneous network by connecting circRNA-circRNA, disease-disease and circRNA-disease through edges weighted by the matrices *CS*, *DS* and *CD*, respectively. Besides, they construct circRNA graph and disease graph to inspect the geometrical structure of circRNA data and disease data. The weight matrix ${W}_{cg}$ of circRNA graph is formulated as follows:

(43) \begin{equation*} {W}_{cg}\left(i,j\right)={W}_c\left(i,j\right)\cdot CS\left(i,j\right) \end{equation*}

(44) \begin{equation*} {W}_c\left(i,j\right)=\left\{\begin{array}{@{}l}1,\kern0.7em \mathrm{if}\kern0.4em {c}_i\in{C}_k\kern0.3em \mathrm{and}\kern0.3em {c}_j\in{C}_k\\{}0,\kern3.999998em \mathrm{otherwise}\end{array}\right.\kern-4pt \end{equation*}

where ${C}_k$ represents the *k*th cluster obtained by using ClusterONE [105] based on circRNA similarity network. Besides, ${D}_c^{\prime }$ is a diagonal matrix, where ${({D}_c^{\prime})}_{ii}={\sum}_j{W}_{cg}(i,j)$. The matrix ${L}_c={D}_c^{\prime }-{W}_{cg}$ denotes the graph Laplacian matrix of circRNA graph. Similarly, the graph Laplacian matrix ${L}_d$ of disease graph can be obtained. Then, to obtain the low-rank feature matrices of circRNAs and diseases, namely *P* and *Q*, which can be used for predicting circRNA-disease associations, they formulate the weighted dual-manifold regularization learning-based calculation model of MRLDC as follows:

(45) \begin{equation*} {\displaystyle \begin{array}{c}\underset{P,Q\ge 0}{\mathit{\min}}\ f\left(P,Q\right)={\left\Vert I\ast \left( CD- PQ\right)\right\Vert}_F^2+{\lambda}_1 Tr\left({P}^T{L}_cP\right)\\{}+{\lambda}_2 Tr\left({QL}_d{Q}^T\right)+{\lambda}_3{\left\Vert{PP}^T- CS\right\Vert}_F^2\\[6pt] {}+{\lambda}_4{\left\Vert{Q}^TQ- DS\right\Vert}_F^2+{\lambda}_5\left({\left\Vert P\right\Vert}_F^2+{\left\Vert Q\right\Vert}_F^2\right)\end{array}} \end{equation*}

where $P$ and $Q$ are the low-rank feature matrices of circRNAs and diseases in the bilayer heterogeneous network, which can be obtained by solving above formula. Besides, $I$ is an indicator weighted matrix where $I(i,j)$ is equal to 1 if circRNA ${c}_i$ is associated with disease ${d}_j$, otherwise $I(i,j)=0$. In addition, ${\lambda}_1,{\lambda}_2,{\lambda}_3,\kern0.35em {\lambda}_4$ and ${\lambda}_5$ are regulation parameters. The second item and the third item in above formula are the manifold regularization terms of circRNA and disease space, respectively. The fourth item (fifth item) is utilized to achieve the purpose that the similarity of circRNAs (diseases) should approximate the inner product of their feature vectors. The last item is to ensure the smoothness of $P$ and $Q$. Next, the Lagrange multiplier method is employed to optimize above objective function and the following updating rules can be obtained:

(46) \begin{equation*} { \begin{array}{c}{P}_{ik}\leftarrow{P}_{ik}\displaystyle\frac{\left({I}_{i\cdot}\ast{CD}_{i\cdot}\right){\left({Q}^T\right)}_{\cdot k}+{\lambda}_1{\left({W}_{cg}P\right)}_{ik}+0.5{\lambda}_3{\left({CS}^TP\right)}_{ik}}{\left({I}_{i\cdot}\ast \left({P}_{i\cdot }Q\right)\right){\left({Q}^T\right)}_{\cdot k}+{\lambda}_1{\left({D}_c^{\prime }P\right)}_{ik}+U}\\{}\left(U=0.5{\lambda}_3{\left({PP}^TP\right)}_{ik}+{\lambda}_5{P}_{ik}\right)\end{array}} \end{equation*}

(47) \begin{equation*} { \begin{array}{c}{Q}_{kj}\leftarrow{Q}_{kj}\displaystyle\frac{{\left({P}^T\right)}_{k\cdot}\left({I}_{\cdot j}\ast{CD}_{\cdot j}\right)+{\lambda}_2{\left({QW}_{dg}\right)}_{kj}+0.5{\lambda}_4{\left({QDS}^T\right)}_{kj}}{{\left({P}^T\right)}_{k\cdot}\left({I}_{\cdot j}\ast \left({PQ}_{\cdot j}\right)\right)+{\lambda}_2{\left(Q{D}_d^{\prime}\right)}_{kj}+V}\\{}\left(V=0.5{\lambda}_4{\left({QQ}^TQ\right)}_{kj}+{\lambda}_5{P}_{jk}\right)\end{array}} \end{equation*}

Finally, the predicted circRNA-disease association matrix $AS= PQ$. The parameters in MRLDC are hard to select. Besides, MRLDC is inappropriate for new disease without any observed associations.

#### iCircDA-MF

**Figure 9:**
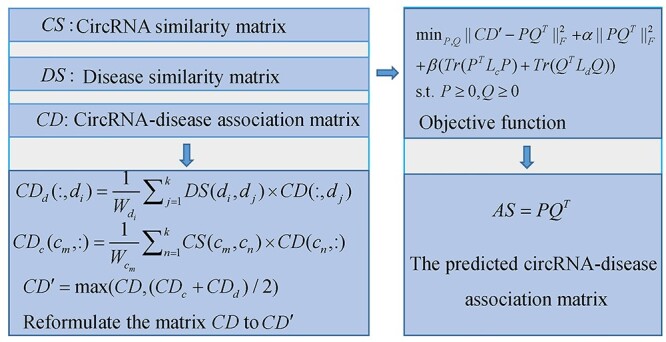
The flowchart of iCircDA-MF to identify circRNA-disease associations based on matrix factorization.

Wei *et al.* [106] proposed a calculation method (see Figure 9) to identify CircRNA-Disease Associations based on Matrix Factorization (iCircDA-MF). In the model of iCircDA-MF, the authors first construct circRNA similarity matrix $CS$ by integrating circRNA GIP kernel similarity and circRNA-related gene-based similarity, and disease similarity matrix $DS$ by integrating disease GIP kernel similarity and disease semantic similarity. Besides, the collected circRNA-disease associations are denoted by the matrix *CD*. However, many false negative associations are assigned as zero in *CD*. To reduce the noise, the authors reformulate the matrix *CD* to ${CD}_d$ and ${CD}_c$ from the vertical direction and the horizontal direction by utilizing the interaction profiles of top-*k* neighbors of investigated disease and circRNA as follows:

(48) \begin{equation*} {CD}_d\left(:,{d}_i\right)=\frac{1}{W_{d_i}}\sum_{j=1}^k DS\left({d}_i,{d}_j\right)\times CD\left(:,{d}_j\right) \end{equation*}

(49) \begin{equation*} {CD}_c\left({c}_m,:\right)=\frac{1}{W_{c_m}}\sum_{n=1}^k CS\left({c}_m,{c}_n\right)\times CD\left({c}_n,:\right) \end{equation*}

where ${CD}_d(:,{d}_i)$ and $CD(:,{d}_j)$ are the *i*th column of ${CD}_d$ and the *j*th column of $CD$. Besides, ${W}_{d_i}={\sum}_{1\le j\le k} DS({d}_i,{d}_j)$. In addition, ${CD}_c({c}_m,;)$ and $CD({c}_n,:)$ denote the *m*th row of ${CD}_c$ and the *n*th row of $CD$. Moreover, ${W}_{c_m}={\sum}_{1\le n\le k} CS({c}_m,{c}_n)$. The final reformulated circRNA-disease association matrix is as follows:

(50) \begin{equation*} C{D}^{\prime }=\max \left( CD,\left({CD}_c+{CD}_d\right)/2\right) \end{equation*}

Next, matrix factorization method is utilized to predict potential circRNA-disease associations, which can be formulated as follows:

(51) \begin{equation*} {\min}_{P\ge 0,Q\ge 0}{\left\Vert C{D}^{\prime }-{PQ}^T\right\Vert}_F^2+\alpha{\left\Vert{PQ}^T\right\Vert}_F^2+\beta \left( Tr\left({P}^T{L}_cP\right)+ Tr\left({Q}^T{L}_dQ\right)\right) \end{equation*}

where *P* and *Q* represent two low-dimension feature matrices of circRNAs and diseases, respectively. In addition, ${L}_c={D}_c^{\prime }- CS$ and ${L}_d={D}_d^{\prime }- DS$ are two graph Laplacian matrices of circRNA and disease space. Here, ${D}_c^{\prime }$ and ${D}_d^{\prime }$ are two diagonal matrices, where ${D}_c^{\prime}(i,i)={\sum}_j CS(i,j)$ and ${D}_d^{\prime}(i,i)={\sum}_j DS(i,j)$. The first item in Eq. (51) is the loss function of matrix factorization method. The second item is used to avoid overfitting and ensure the smoothness of circRNA and disease space. Besides, the last item can restrict the geometrical structure of target space and reduce noise [107, 108]. In addition, $\alpha$ and $\beta$ are regulation parameters.

Finally, the predicted circRNA-disease association matrix $AS$ can be calculated as $AS={PQ}^T$ after solving Eq. (51). This work can effectively deal with noise data.

#### GMCDA

Xiao *et al.* [109] designed a Graph-based Multi-label learning for CircRNA-Disease Association prediction (GMCDA). The integrated similarity matrices of *CS* and *DS* are obtained by fusing directed acyclic graphs of diseases and circRNA-disease associations. The authors aim to generate an expected association matrix *AS* to restore the missing values in the original circRNA-disease association matrix *CD*. To achieve the aim, the multi-label learning-based framework is proposed and formulated by an objective function with three constraints as follows:

(52) \begin{equation*} {\displaystyle \begin{array}{l}\underset{AS\ge 0}{\min }{\left\Vert I\ast \left( AS- CD\right)\right\Vert}_F^2\\{}\kern2.2em +\lambda \left({\left\Vert AS\times CS\times{AS}^T- CS\right\Vert}_F^2+{\left\Vert{AS}^T\times DS\times AS- DS\right\Vert}_F^2\right)\\[6pt] {}\kern2.1em +\gamma \left( Tr\left({AS}^T\times{L}_c\times AS\right)+ Tr\left( AS\times{L}_d\times{AS}^T\right)\right)+\mu{\left\Vert AS\right\Vert}_{1,2}^2\end{array}} \end{equation*}

where *I* is an indicator matrix (*I*=*CD*). Besides, the graph Laplacian matrices of ${L}_c$ and ${L}_d$ can be computed by the same way used in the previous model of MRLDC. In addition, $\lambda$, $\gamma$ and $\mu$ are constants used to control the contributions of different terms. The first item in above formula is the loss function of GMCDA. The second item means that the expected similarity values of circRNA pairs and disease pairs should be approximate to the original similarities. The third item is used to capture geometrical structures of data. The last item is utilized to increase the sparsity of *AS* and reduce noisy. The local optimal solution of this objective function can be obtained by an iterative method.

#### iCDA-CMG

Xiao *et al.* [110] proposed the algorithm of identifying CircRNA-Disease Associations by using Collective Matrix completion with Graph learning (iCDA-CMG). First, the circRNA similarity matrix *CS* is obtained based on circRNA-disease association information. Besides, the disease similarity matrix *DS* fuses the data of directed acyclic graphs of diseases and circRNA-disease associations. Then, the DWNN method, in the same way as that used in the model of iCircDA-MF, is adopt to reconstruct circRNA-disease association matrix *CD* to the matrix $C{D}^{\prime }$.

Next, the similarity matrices of *CS* and *DS* are reconstructed to the sparse similarity matrices of $C{S}^{\prime }$ and $D{S}^{\prime }$ by utilizing the structure information of circRNA graph (circRNA similarity network) and disease graph (disease similarity network). Subsequently, the objective function of iCDA-CGM is formulated to obtain the latent circRNA feature matrix $P\in{R}^{K\times{N}_c}$ and the latent disease feature matrix $Q\in{R}^{K\times{N}_d}$ as follows:

(53) \begin{equation*} \displaystyle \begin{array}{c}\underset{P\ge 0,Q\ge 0}{\mathit{\min}}{\left\Vert C{D}^{\prime }-{P}^TQ\right\Vert}_F^2+{\lambda}_c\sum\limits_{i,j=1}^{N_c}{\left\Vert P\left(:,i\right)-P(:,j)\right\Vert}_F^2C{S}_{i,j}^{\prime}\\{}+{\lambda}_d\sum\limits_{i,j=1}^{N_d}{\left\Vert Q\left(:,i\right)-Q(:,j)\right\Vert}_F^2D{S}_{i,j}^{\prime}\\{}+{\delta}_c\sum\limits_{i=1}^{N_c}{\left\Vert P\left(:,i\right)\right\Vert}_1^2+{\delta}_d\sum_{i=1}^{N_d}{\left\Vert Q(:,i)\right\Vert}_1^2\end{array} \end{equation*}

where the parameters of ${\lambda}_c$, ${\lambda}_d$, ${\delta}_c$ and ${\delta}_d$ are utilized to control the contributions of different regulation terms. The first item in above formula is the loss function of collective matrix completion. The second item (third item) is employed to achieve the purpose that the latent feature vectors of similar circRNAs (diseases) should be similar. The last two items are used to ensure the sparsity of *P* and *Q*. Finally, an alternating method with Lagrange multipliers is used to solve the objective function, and the predicted circRNA-disease association matrix is $AS={P}^TQ$.

#### NMFIBAC

Wang *et al*. [111] developed a Non-negative Matrix Factorization algorithm (NMF)-based model to Identify Breast cancer Associated CircRNAs (NMFIBAC), which integrated multiple biological data including mRNA, miRNA, circRNA and pathway-related data. Firstly, they search DE circRNAs and miRNAs from RNA-seq data involving disease samples and normal samples. Then, they construct circRNA-mRNA association matrix ${X}_1$ based on DE circRNAs and co-expressed mRNAs, miRNA-mRNA association matrix ${X}_2$ based on DE miRNAs and miRNA target genes, as well as pathway-mRNA association matrix ${X}_3$. Subsequently, NMF algorithm is utilized to establish *K* circRNA modules by the following objective function *F*:

(54) \begin{equation*} F\left(W,H\right)=\sum_{I=1}^3\left\Vert{X}_I-{WH}_I\right\Vert \end{equation*}

where *W* is a matrix with the size of $M\times K$ (*M* denotes the number of mRNAs) representing the basis vector. In addition, the matrix ${H}_I(I\in (1,2,3))$ denotes the coefficient vector. After solving the objective function, the matrix $W$ and ${H}_I(I\in (1,2,3))$ are utilized to determine the members (including miRNAs, mRNAs, circRNAs and pathways) of the *K* circRNA modules based on a previous method [112]. Finally, in each module, circRNAs connecting with more than four members are considered to be associated with breast cancer.

#### SIMCCDA

**Figure 10:**
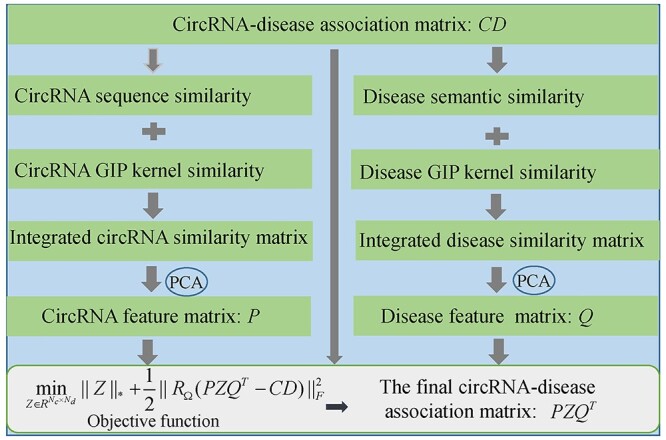
The framework of SIMCCDA for circRNA-disease association prediction based on inductive matrix completion.

Li *et al.* [113] raised a model (see Figure 10) of Speedup Inductive Matrix Completion for CircRNA-Disease Association prediction (SIMCCDA). In SIMCCDA, *CS* and *DS* are calculated by combining circRNA sequence similarity, circRNA GIP kernel similarity, disease semantic similarity and disease GIP kernel similarity. Besides, principal component analysis is utilized to extract primary feature vectors of the matrices *CS* and *DS*. The extracted feature vectors are used to construct the circRNA feature matrix *P* and disease feature matrix *Q*. The objective function of inductive matrix completion can be defined as

(55) \begin{equation*} \underset{Z\in{R}^{N_c\times{N}_d}}{\min }{\left\Vert Z\right\Vert}_{\ast }+\frac{1}{2}{\left\Vert{R}_{\Omega}\left({PZQ}^T- CD\right)\right\Vert}_F^2 \end{equation*}

where *Z* is the target matrix to complete *CD* and ${\Vert \cdot \Vert}_{\ast }$ denotes the nuclear norm. Besides, ${PZQ}^T$ is the final circRNA-disease association matrix. In addition, $\Omega$ denotes known association sets. The first item in Eq. (55) is the constraint of low rank. The second item is employed to cater to the hypothesis that the row (or column) vectors in *CD* are located in the subspace spanned by the column vectors in *Q* (or *P*). The solution of *Z* can be obtained by using an accelerated proximal gradient algorithm [114].

#### PreCDA

**Figure 11:**
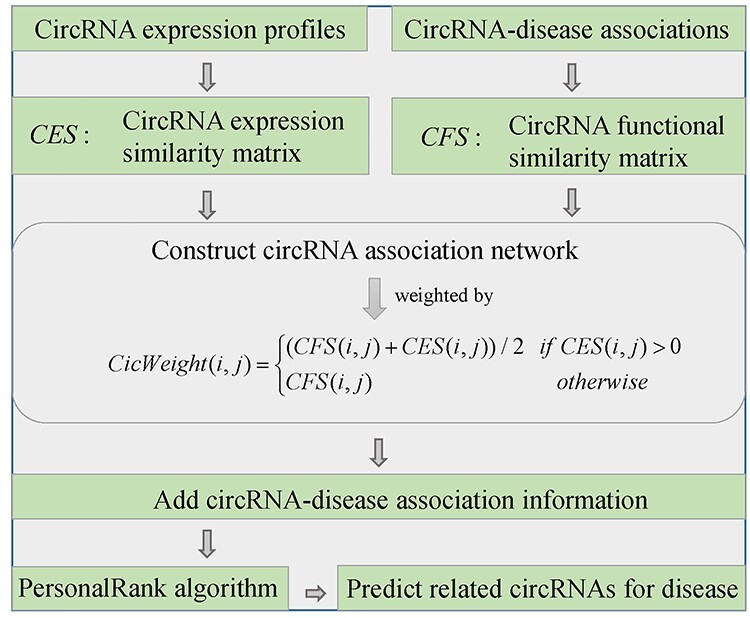
The workflow of PreCDA to infer underling circRNA-disease associations based on PersonalRank algorithm.

Wang *et al*. [115] developed a calculation model named PreCDA to infer underling circRNA-disease associations (see Figure 11). They compute circRNA expression similarity matrix $CES$ by Spearman correlation coefficient based on circRNA expression profile in 78 human cell types or tissues. Besides, the circRNA functional similarity matrix $CFS$ is calculated based on known circRNA-disease associations. Then, they construct a circRNA association network, where the weight between circRNA ${c}_i$ and ${c}_j$ is defined as

(56) \begin{equation*} \mathrm{CicWeight}\left(i,j\right)=\left\{\begin{array}{@{}l}\left( CFS\left(i,j\right)+ CES\left(i,j\right)\right)/2\kern1em \mathrm{if}\kern0.3em CES\left(i,j\right)>0\\{} CFS\left(i,j\right)\kern11.10001em \mathrm{otherwise}\end{array}\right.\kern-4pt \end{equation*}

To infer potential disease-associated circRNAs, the information of circRNA-disease associations is introduced into the circRNA association network. Based on the new network composed of circRNAs and diseases, PersonalRank algorithm is employed to identify disease-related circRNAs. Specifically, $PR(i)$ is used to denote the possibility value that node *i* is accessed. In the beginning, $PR(i)$ is equal to 1 if the node *i* is the target disease node *t*, otherwise 0. Then, the target node *t* randomly moves to neighbor nodes. In each move, the probability of returning to node *t* is $(1-\alpha )$. The following formula is defined to update $PR(i)$ after each move:

(57) \begin{equation*} PR(i)=\left(1-\alpha \right){r}_i+\alpha \sum \limits_{j\in \mathrm{in}(i)}\frac{PR(j)}{\mid \mathrm{out}(j)\mid } \end{equation*}

(58) \begin{equation*} {r}_i=\left\{\begin{array}{@{}l}1\kern2em \mathrm{if}\kern0.5em i=t\\{}0\kern1.8em \mathrm{if}\kern0.3em i\ne t\end{array}\right.\kern-4pt \end{equation*}

where $\mathrm{in}(i)$ and $\mathrm{out}(j)$ are the in-degree of node *i* and out-degree of node *j*, respectively; *d* is the transfer probability; *t* denotes the target node. After enough moves, the possibility value that node *i* is accessed will be stable. Finally, the probability value that a circRNA node is accessed can be used as the association score between the target disease *t* and this circRNA. The main limitation of PreCDA lies in the invalid application for disease without any known related circRNAs.

#### ICFCDA

**Figure 12:**
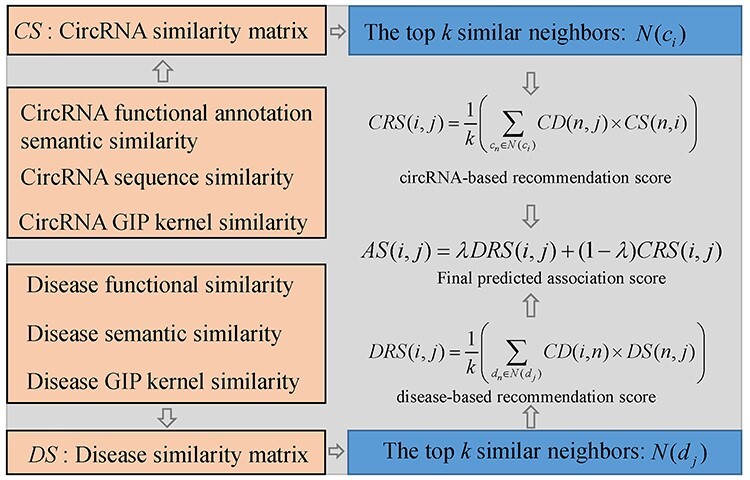
The workflow of ICFCDA to predict circRNA-disease associations based on improved collaboration filtering recommendation system.

Lei *et al*. [116] raised an improved collaboration filtering recommendation system-based model named ICFCDA to predict circRNA-disease associations (see Figure 12). They construct circRNA similarity matrix *CS* by integrating circRNA functional annotation semantic similarity, circRNA sequence similarity as well as circRNA GIP kernel similarity. Besides, the disease similarity matrix *DS* can be obtained by integrating disease functional similarity, disease semantic similarity and disease GIP kernel similarity. To calculate recommendation score between circRNA ${c}_i$ and disease ${d}_j$, the top *k* similar neighbors $N({c}_i)$ of ${c}_i$ and the top *k* similar neighbors $N({d}_j)$ of disease ${d}_j$ are selected according to similarity matrices of circRNA and disease. Then, circRNA-based recommendation score between ${c}_i$ and ${d}_j$ can be computed based on the matrices of *CD* and *CS* as follows:

(59) \begin{equation*} CRS\left(i,j\right)=\frac{1}{k}\left(\sum \limits_{c_n\in N\left({c}_i\right)} CD\left(n,j\right)\times CS(n,i)\right) \end{equation*}

Similarly, disease-based recommendation score between ${c}_i$ and ${d}_j$ is defined as follows:

(60) \begin{equation*} DRS\left(i,j\right)=\frac{1}{k}\left(\sum \limits_{d_n\in N\left({d}_j\right)} CD\left(i,n\right)\times DS(n,j)\right) \end{equation*}

Finally, the two recommendation scores are integrated as the predicted association score between ${c}_i$ and ${d}_j$ as follows:

(61) \begin{equation*} AS\left(i,j\right)=\lambda DRS\left(i,j\right)+\left(1-\lambda \right) CRS\left(i,j\right) \end{equation*}

where the parameter $\lambda$ is a balance factor.

### The second type of machine learning-based models

#### RWRKNN

**Figure 13:**
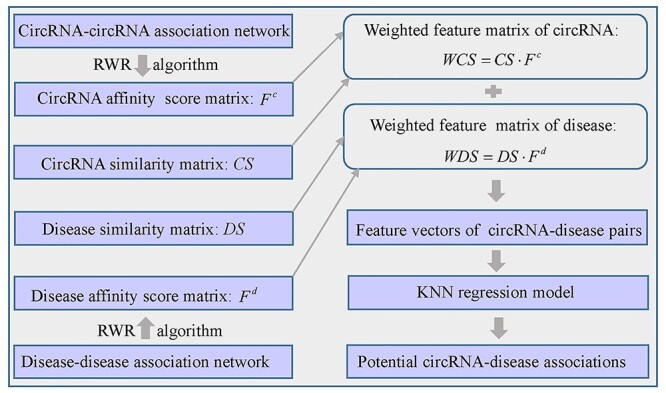
The workflow of RWRKNN to predict novel circRNA-disease associations based on random walk with restart and KNN.

Lei *et al.* [117] put forward a method named Random Walk with Restart and KNNs (RWRKNN) (see Figure 13) to predict novel circRNA-disease associations. Firstly, they construct disease similarity matrix *DS* by integrating disease semantic similarity and GIP kernel similarity, and circRNA similarity matrix *CS* by integrating circRNA functional similarity and GIP kernel similarity. The matrices of *DS* and *CS* are considered to be the feature matrices of disease and circRNA. Secondly, the matrices of *DA* and *CA* are utilized to represent disease-disease association network and circRNA-circRNA association network, respectively. These two matrices can be defined as follows:

(62) \begin{equation*} DA\left(i,j\right)=\left\{\begin{array}{@{}l}1\kern1.4em \mathrm{if}\kern0.4em DS\left(i,j\right)\ge \alpha \\{}0\kern1.1em \mathrm{otherwise}\end{array}\right. \end{equation*}

(63) \begin{equation*} CA\left(i,j\right)=\left\{\begin{array}{@{}l}1\kern1.4em \mathrm{if}\kern0.4em CS\left(i,j\right)\ge \beta \\{}0\kern1.1em \mathrm{otherwise}\end{array}\right.\kern-4pt \end{equation*}

where $\alpha$ and $\beta$ are different threshold values.

Thirdly, the affinity scores between a disease (circRNA) node and all disease (circRNA) nodes can be calculated by utilizing RWR algorithm on the disease-disease (circRNA-circRNA) association network. The matrices of ${F}^c$ and ${F}^d$ denote the affinity scores for circRNA and disease, respectively. Next, the weighted feature matrices of circRNA and disease, namely *WCS* and *WDS*, are defined as follows:

(64) \begin{equation*} WCS= CS\cdot{F}^c \end{equation*}

(65) \begin{equation*} WDS= DS\cdot{F}^d \end{equation*}

The feature vectors of circRNA-disease pairs can be obtained by splicing the row vector of $WCS$ and $WDS$. Finally, KNN regression model is adopted to predict potential circRNA-disease associations.

#### iCDA-CGR

Zheng *et al.* [118] proposed the method of identification of CircRNA-Disease Associations based on Chaos Game Representation (iCDA-CGP). The matrix of $DS$ is constructed by integrating disease semantic similarity and GIP kernel similarity, while the matrix $CS$ is constructed by integrating circRNA-related gene-based similarity, circRNA sequence-based similarity and circRNA GIP kernel similarity. The model of iCDA-CGP can be roughly divided into three steps. First of all, they construct training sample set including the same number of positive and negative samples. The positive samples are gathered from benchmark database of circRNA-disease associations, while the negative samples are selected from unlabeled circRNA-disease pairs. Secondly, the descriptor of each circRNA-disease pair in the training sample set can be formed based on the matrices of *CS* and *DS*

(66) \begin{equation*} F\left({d}_i,{c}_j\right)=\left( DS\left(i,:\right), CS\left(j,:\right)\right) \end{equation*}

where $F({d}_i,{c}_j)$ denotes the descriptor of the pair of ${d}_i$ and ${c}_j$. Besides, $DS(i,:)$ and $CS(j,:)$ are the *i*th row of $DS$ and the *j*th row of $CS$. Finally, based on SVM, the descriptors of training samples are utilized to train prediction model which is used to infer novel circRNA-disease associations. The model of iCDA-CGP has one main limitation, that is the negative samples used in the model are not reliable.

#### GBDTCDA

**Figure 14:**
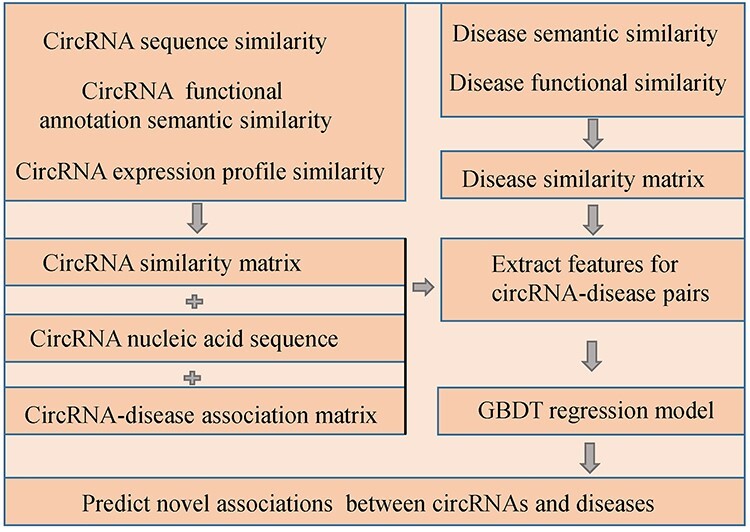
The workflow of GBDTCDA to predict potential circRNA-disease associations based on GBDT algorithm.

Lei *et al*. [119] developed a prediction model of GBDT with multiple biological data to predict CircRNA-Disease Association (GBDTCDA) (see Figure 14). Specifically, they compute circRNA sequence similarity, circRNA functional annotation semantic similarity as well as circRNA expression profile similarity, and combine them into the matrix *CD* by a similarity network fusion algorithm [120]. In addition, they integrate disease semantic and functional similarity as the matrix *DS* by endowing different weights for the two types of similarity. Secondly, four types of features of each circRNA-disease pair are extracted from the data of collected circRNA-disease associations, integrated similarity of circRNAs and diseases as well as circRNA nucleic acid sequence. The feature vector of the pair of circRNA ${c}_i$ and disease ${d}_j$ can be denoted as follows:

(67) \begin{equation*} F\left({c}_i,{d}_j\right)=\left[{F}_1\left({c}_i,{d}_j\right),{F}_2\left({c}_i,{d}_j\right),{F}_3\left({c}_i,{d}_j\right),{F}_4\left({c}_i,{d}_j\right)\right] \end{equation*}

where ${F}_i$ represents the *i*th type of features. Finally, they utilize GBDT regression to train the training samples and obtain predictive model for potential circRNA-disease association identification. In GBDTCDA, the authors make full use of multiple biological data and extract various kind of features, which facilitates the reliable performance of GBDTCDA.

#### DFPUCDA

Zeng *et al*. [121] raised a computational model of DF combined with Positive-Unlabeled learning based CircRNA-Disease Association prediction (DFPUCDA). In the first step of DFPUCDA, the authors construct a heterogeneous biological network, which contains a disease similarity network, a miRNA functional similarity network, a circRNA co-expression network, a miRNA-circRNA interaction network and a miRNA-disease association network. Then, they extract 24 meta-path-based features to represent circRNA-disease samples by PathCount and RandomWalk measures [122, 123]. Next, a positive-unlabeled learning algorithm is exploited to select reliable negative samples from unlabeled samples. Subsequently, DF algorithm is employed to train a classifier with collected positive samples and reliable negative samples. Finally, they utilize the classifier to infer positive circRNA-disease samples. It is difficult to obtain negative circRNA-disease samples and the number of positive samples is far less than that of unlabeled samples. In DFPUCDA, the positive-unlabeled algorithm can make full use of the information of unlabeled samples and solve the problem of data imbalance to some extent.

#### CNNCDA

Wang *et al*. [124] put forward a CNN-based method to predict CircRNA-Disease Associations (CNNCDA). Firstly, they construct the matrix $DS$ through merging disease semantic similarity and disease GIP kernel similarity. Besides, the matrix $CS$ is constructed based on circRNA GIP kernel similarity. Secondly, the authors define the circRNA-disease fusion descriptor $F({c}_i,{d}_j)$ between circRNA ${c}_i$ and disease ${d}_j$ as follows:

(68) \begin{equation*} F\left({c}_i,{d}_j\right)=\left[ CS\left(i,:\right), DS\left(j,:\right)\right] \end{equation*}

where $CS(i,:)$ and $DS(j,:)$ denote the *i*th row and *j*th row of $CS$ and $DS$, respectively.

Next, CNN, composed of input layer, convolution layer, subsampling layer, full connection layer and the output layer, is utilized to extract hidden deep features from circRNA-disease fusion descriptor. Finally, the extreme learning machine algorithm [125, 126] is used to train prediction model based on positive circRNA-disease samples and negative samples. However, the circRNA similarity is computed only based on known circRNA-disease associations, which would reduce the prediction performance.

#### GCNCDA

**Figure 15:**
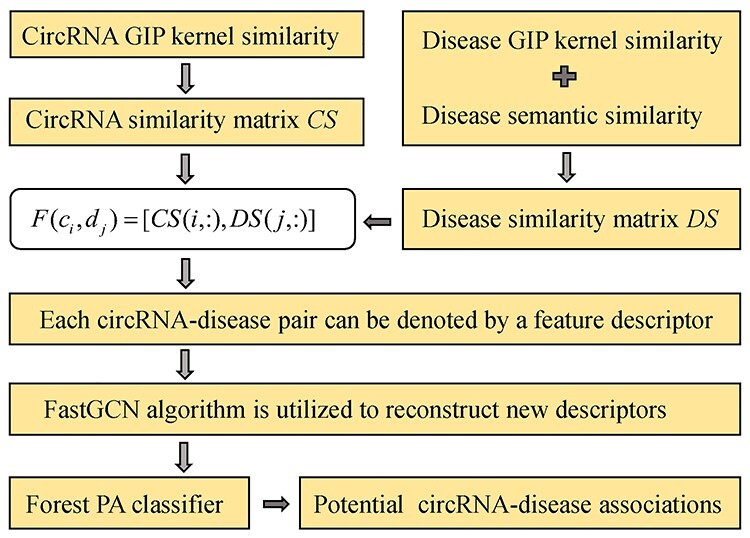
The flow diagram of GCNMDA to predict potential circRNA-disease associations based on Graph Convolutional Network.

Wang *et al.* [127] further proposed a Graph Convolutional Network-based algorithm to infer CircRNA-Disease Associations (GCNCDA) whose flow diagram is shown in Figure 15. Firstly, the circRNA similarity matrix *CS* is constructed based on circRNA GIP kernel similarity, and the disease similarity matrix *DS* is constructed based on disease GIP kernel similarity and disease semantic similarity. Secondly, each circRNA-disease pair can be denoted by a feature descriptor which can be obtained in the same way as that in CNNCDA (i.e. Eq. (68)). Then, the Fast learning with Graph Convolutional Networks (FastGCN) [128] is utilized to further extract high-level features from original feature descriptors to construct new descriptors. Compared with GCN, FastGCN can make the training process more efficient. Next, the Forest by Penalizing Attributes (Forest PA) algorithm [129] is used to train classifier. Forest PA generates the training data set for trees by bootstrap sampling. The decision trees are built by using an improved CART algorithm [130]. The only difference between original CART algorithm and the improved CART algorithm is that the merit values is employed to instead of classification capacities (e.g. Gini Index) to select splitting attributes. Finally, the Forest PA classifier can be used to predict potential circRNA-disease associations.

#### AE-DNN

Deepthi *et al.* [131] devised an ensemble method to predict circRNA-disease associations based on AutoEncoder and DNN (AE-DNN). First, the circRNA similarity matrix is constructed by integrating circRNA sequence similarity and circRNA GIP similarity, while the disease similarity matrix $DS$ is computed by integrating disease semantic similarity as well as disease GIP similarity. Then, they construct training sample set which contains both positive and negative samples. The positive samples are obtained from the CircR2Disease database and the negative samples are randomly selected from unlabeled circRNA-disease pairs. For each training sample $({c}_i,{d}_j)$, the feature vector is the splicing of the vectors of $CS(i,:)$ and $DS(j,:)$. Next, the autoencoder consisting of encoder and decoder is utilized to extract the high-level features and reduce the dimension of feature vectors. Autoencoder [132] is a special neural network structure, which can learn the latent features of input data. Finally, the high-level feature vectors of training samples are used to train a three-layer feed-forward DNN. After training, the DNN can predict association probability for unlabeled circRNA-disease pair.

#### AE-RF

**Figure 16:**
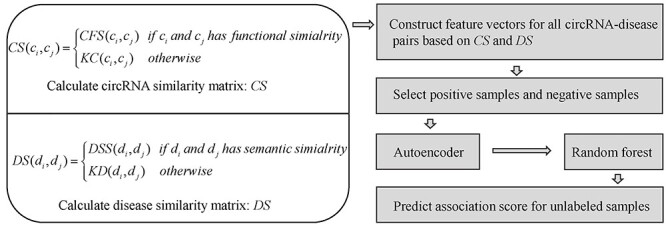
The flow diagram of AE-RF to predict potential circRNA-disease associations based on AutoEnconder and RF.

Deepthi *et al.* [133] proposed an ensemble method of circRNA-disease association prediction based on a deep AntoEncoder and RF classifier (AE-RF) whose flow diagram is shown in Figure 16. They first construct circRNA similarity matrix *CS* and disease similarity matrix *DS* by combing multiple types of similarity of circRNA and disease as follows:

(69) \begin{equation*} CS\left({c}_i,{c}_j\right)=\left\{\begin{array}{@{}l} CFS\left({c}_i,{c}_j\right)\kern1em \mathrm{if}\kern0.2em {c}_i\kern0.3em \mathrm{and}\kern0.3em {c}_j\kern0.3em \mathrm{has}\kern0.3em \mathrm{functional}\kern0.3em \mathrm{simialrity}\\{} KC\left({c}_i,{c}_j\right)\kern1.6em \mathrm{otherwise}\end{array}\right. \end{equation*}

(70) \begin{equation*} DS\left({d}_i,{d}_j\right)=\left\{\begin{array}{@{}l} DSS\left({d}_i,{d}_j\right)\kern1em \mathrm{if}\kern0.2em {d}_i\kern0.3em \mathrm{and}\kern0.3em {d}_j\kern0.3em \mathrm{has}\kern0.3em \mathrm{semantic}\kern0.3em \mathrm{simialrity}\\{} KD\left({d}_i,{d}_j\right)\kern1.6em \mathrm{otherwise}\end{array}\right.\kern-4pt \end{equation*}

Then, the feature vector of circRNA-disease pair $({c}_i,{d}_j)$ is constructed by splicing the vector $CS({c}_i,:)$ and vector $DS({d}_j,:)$. Next, the training set consisting of equal positive and negative samples is utilized to train an autoencoder which is also used in the prediction model of AE-DNN. After training, the autoencoder can be used to reconstructed the feature vectors of samples in training set and remaining unlabeled circRNA-disease pairs. Subsequently, the training samples are utilized to train the RF classifier. The trained classifier can be used to predict association score for unlabeled samples. The innovative of this study lies in the combined application of autoencoder and RF where autoencoder can help reduce noise data and extract high-level features, while RF has good generalization ability. However, the false negative problem of randomly selected negative samples still exists.

## Algorithm evaluation methods

To evaluate the predictive performance of computational models, researchers usually report their AUC values based on distinct cross validation including LOOCV, 5-fold and 10-fold cross validation (collectively called *K*-fold cross-validation). LOOCV and *K*-fold cross validation have been widely utilized to evaluate the performance of not only the circRNA-disease association prediction models but also other biological association prediction models, such as miRNA-disease association prediction models [92, 99, 134], lncRNA-disease association prediction models [95, 135], lncRNA-miRNA interaction and lncRNA-protein interaction prediction models [136–138]. In this section, we will introduce LOOCV and *K*-fold cross validation in detail. In addition to cross validation, we also introduced two types of case studies, which have been frequently utilized to evaluate the prediction performance of different circRNA-disease association prediction algorithms.

### LOOCV

In the process of LOOCV, each known circRNA-disease association is left out as the test sample in turn, and the remaining known associations are adopted as training samples. In addition, all unknown circRNA-disease pairs are candidate samples. Specifically, the prediction model based on the training samples can score for the investigated test sample and all candidate samples. Then, the test sample and candidate samples are ranked in descending order according to their association scores. Above process is repeated until every known circRNA-disease association is tested. According to the results of LOOCV, true positive rate (*TPR*) and false positive rate (*FPR*) can be calculated as follows:

(71) \begin{equation*} TPR=\frac{TP}{TP+ FN} \end{equation*}

(72) \begin{equation*} FPR=\frac{FP}{FP+ TN} \end{equation*}

where *TP* denotes the number of true positive samples which are test samples ranked higher than the given threshold; *FN* denotes the number of false negative samples, which are test samples ranked lower than the given threshold. In addition, *FP* represents the number of false positive samples, which are candidate samples ranked higher than the given threshold; *TN* represents the number of true negative sample, which are candidate samples ranked lower than the given threshold. The ROC (receiver operating characteristic) curve can be drawn by plotting the *TPR* against the *FPR* under a series of thresholds. Furthermore, the value of AUC can demonstrate the performance of prediction model and the higher the AUC, the better the prediction performance of the model.

### K-fold cross validation

In *K*-fold cross validation, all known circRNA-disease associations are divided into *K* subsets with the same size. Then, one of the *K* subsets is left out as the test set and the remaining *K* − 1 subsets are utilized as training set to train the prediction model. All unknown circRNA-disease pairs are candidate samples. The trained prediction model can score for the samples in the test set and candidate samples. Next, each sample in the test set is ranked with the candidate samples in descending order according to their association scores. When all the *K* subsets have been tested, the ROC curve and AUC value can be drawn and calculated in the same way used in the LOOCV.

### Case study

Usually, one or several diseases would be investigated in case study. In addition, the types of case studies are also diverse. In the following, we will introduce two common types of case studies utilized to evaluate predictive performance of circRNA-disease association prediction model. The first type of case study aims to assess the prediction ability of calculation model in identifying novel circRNA-disease relationships [85, 102]. Specifically, the trained prediction model is used to compute the association scores for candidate samples involving investigated disease. Then, the result of case study for investigated disease can be obtained by inspecting how many associations in the top-*M* predicted results have been confirmed by other database or literature. The second type of case study aims to evaluate the prediction ability of calculation model in predicting associated circRNAs for novel disease without any known related circRNAs [86, 117]. To be more specific, the association information involving an investigated disease is removed from training sample set. Then, the trained model is utilized to infer associated circRNAs for this investigated disease. Finally, researches observe how many circRNAs in the top-ranked predictions have been confirmed by database or literature.

## Discussion and conclusion

CircRNAs have caught much attention from scientists. More and more circRNAs were discovered by biological experiments and bioinformatics methods. Later, researchers found that circRNAs have important biological functions including acting as miRNA sponges, regulating the expression of parental genes as well as competing with pre-mRNA splicing. In addition, many experimental evidences indicate that circRNAs have close relationships with complex human diseases. The occurrence and development of many complex diseases are usually accompanied by abnormal expression of circRNA. Thus, studying associations between circRNAs and diseases could promote the understanding of the functions of circRNAs and the pathogenesis of complex diseases, which would further provide new ideas and strategies for detection, diagnosis and treatment of complex diseases. Identifying novel circRNA-disease associations is a critical step. However, it is inefficient to discover novel associations by traditionally experimental methods. Fortunately, massive biological data about circRNAs and circRNA-disease associations have been accumulated after conducting various biological experiments and RNA sequencing. Therefore, researchers have proposed effective computational methods to predict novel circRNA-disease relationships by mining useful information from biological data such as circRNA sequence, circRNA expression profile, disease directed acyclic graph, circRNA-gene interaction, disease-gene association and circRNA-disease association.

In this review, we first briefly summarized the general concepts and classification of circRNAs. Then, we introduced some common functions of circRNAs and associations between circRNAs and several important human diseases, since circRNAs may be a novel classes of biomarkers of complex diseases. Next, we presented two types of databases which can provide biological data about circRNAs and circRNA-disease associations. Proper application of these databases can promote the research of circRNA function and identification of novel circRNA-disease associations. Subsequently, we introduced 27 computational models for inferring novel circRNA-disease associations. According to the core algorithms used in these models, we divided the computational models into two classes, namely network algorithm-based models and machine learning-based models. Finally, we summarized several common measures for performance evaluation of circRNA-disease association prediction models.

In the following, we will discuss the advantages and limitations of aforementioned two types of computational models. First of all, in the network algorithm-based models, it is a key step to construct the circRNA-disease associations network, circRNA similarity network and disease similarity network. Generally, circRNA-circRNA similarity can be calculated based on circRNA sequences, circRNA-related genes, expression profiles of circRNAs- and circRNA-related diseases. In addition, disease-disease similarity can be computed based on disease related genes, phenotype descriptions of diseases, directed acyclic graphs of diseases and disease-related circRNAs. The different network algorithms, such as KATZ, label propagation and bipartite network projection, were utilized to infer novel circRNA-disease associations based on these networks. One advantage of network algorithm lies that these models can integrate multiple biological data to construct single layer network or heterogeneous network and make full use of topological information of circRNA-disease network. In addition to circRNA and disease, other biological object can also be introduced into heterogeneous networks. For example, in the model of BRWSP, the authors introduced gene similarity network, gene-disease association network and gene-circRNA interaction network into their constructed heterogeneous network. Another advantage of network algorithm lies in the wide choice for similarity calculation methods. Except for the full use of multiple data, similarity calculation method also plays an important role in network algorithm-based models. For example, in the model of CD-LNLP, the authors utilized LNS measure to calculate circRNA similarity and disease similarity. As a result, CD-LNLP obtains impressive performance even though only circRNA-disease association data are used to calculate similarity. Therefore, reliable similarity calculation method would contribute to the predictive performance of network algorithm-based models. However, most of network algorithm-based models cannot predict associations for diseases without any known related circRNAs. Besides, it is difficult to determine the weights of distinct types of similarity in the process of similarity integration. Therefore, how to construct different circRNA similarity networks and disease similarity networks, and reasonably integrate the similarity from different biological source information is an important topic worthy of further study.

Machine learning-based circRNA-disease association prediction models could be further divided into two classes. Specifically, regularized least squares, logistic regression and manifold regularization learning, matrix decomposition and inductive matrix completion algorithm-based calculation methods belong to the first category, which usually transform the problem of circRNA-disease association prediction into solving diverse optimization models based on circRNA-disease adjacency matrix, circRNA similarity matrix and disease similarity matrix. One advantage of the first class of machine learning-based models is that negative samples are not necessary. Actually, negative circRNA-disease associations are hard to collect due to the fact that experimentally validated negative circRNA-disease relationships are usually not reported in literature or database. Besides, different regulation terms can be added into objective functions of the first types of machine learning-based models. For example, in the models of MRLDC and iCircRA-MF, graph regularization term is introduced into their objective functions to restrict the geometrical structure of target space and reduce noise. However, the parameters in the objective functions are hard to determine. In addition, how to choose suitable optimization algorithm to solve different objective functions is worth considering. In the second type of machine learning-based circRNA-disease association prediction models, the algorithms of KNN, SVM, RF, GBDT, DF, CNN, GNN and DNN are utilized to construct different classifiers. Besides, distinct feature construction methods are employed in the second type of machine learning-based models. One advantage of these models lies that they could make full use of the prior information of known circRNA-disease associations since all know positive samples are utilized to train the prediction models. In addition, most of the second type of machine learning-based models can be employed to predict associated circRNAs for novel disease without any known related circRNAs. However, negative samples are necessary in these prediction models. As mentioned above, negative circRNA-disease samples are difficult to collect and randomly selecting unlabeled samples as negative samples is a common strategy in these models, which would reduce the prediction accuracy to some extent. Furthermore, the second type of machine learning-based models belong to supervised learning models, so the class imbalance problem of circRNA-disease samples is one of main obstacles in these prediction models. Semi-supervised learning methods work well dealing with the class-imbalance data. Therefore, researchers can utilize semi-supervised learning algorithms to establish new prediction models in the future.

Overall, circRNA plays an important role in the development of various complex diseases and is a novel biomarker of complex diseases. Accumulation of experimental data about circRNAs and diseases makes it possible to predict new circRNA-disease associations by computational methods. However, the number of current known circRNA-diseases associations is too less, which limits the predictive accuracy of existing computational models. Thus, collection and accumulation of experimentally verified circRNA-disease associations remains an important mission in the future study. Besides, researchers can consider utilizing the information of other biological objects, such as pathway and protein, to help circRNA-disease association prediction, since biological objects are usually closely interdependent. In terms of calculation model, new effective algorithms should be proposed since the current methods have different limitations. In this paper, we mainly reviewed the research of circRNA-disease association from distinct aspects. Actually, the studies of miRNA-disease association and lncRNA-disease association are also hot research fields [134, 135]. MiRNAs and lncRNAs also play important roles in the occurrence and development of many human diseases. However, the studies of associations between circRNAs, miRNAs, lncRNAs and human diseases were conducted independently. The joint research of associations between circRNAs, miRNAs, lncRNAs and human diseases may be an important future direction. In the end, scientists have demonstrated that non-coding RNA can be of drug targets [101]. Specially, some works have been implemented to identify miRNAs as drug targets [139–141]. CircRNA is also an important class of non-coding RNA. Therefore, identifying circRNAs as drug targets could be a promising future direction.

**Key Points**

- CircRNAs play a growing important role in a large number of life activities and are thus closely related to various human complex diseases.
- Studying associations between circRNAs and diseases could promote the understanding of the functions of circRNAs and the pathogenesis of complex diseases.
- We listed some publicly accessible databases about circRNAs and circRNA-disease associations.
- Computational models could effectively predict potential circRNA-disease associations for further experimental verification, which would save many resources.
- Computational models of circRNA-disease prediction were divided into two categories, namely network algorithm and machine learning-based model.
- We introduced several methods of algorithm evaluation to estimate the predictive performance of calculation models.
- The advantages and limitations of various existing computational models were analyzed.

## Supplementary Material

Supplementary_materials_bbab286

## Data Availability

The source code of SIMCCDA is available at https://github.com/bioinformaticsAHU/SIMCCDA. The source code of PreCDA is available at https://github.com/wyt-nwpu/PreCDA. The source code of DFPUCDA is available at https://github.com/xzenglab/DeepDCR. The source code of AE-RF is available at https://github.com/Deepthi-K523/AE-RF.

## Notes

**Chun-Chun Wang** is a PhD student of the School of Information and Control Engineering, China University of Mining and Technology. His research interests include bioinformatics, complex network algorithm and machine learning.

**Chen-Di Han** is a master student of the School of Information and Control Engineering, China University of Mining and Technology. Her research interests include bioinformatics and deep learning.

**Qi Zhao**, PhD, is a Professor of the School of Computer Science and Software Engineering, University of Science and Technology Liaoning. His research interests include bioinformatics, complex network and machine learning.

**Xing Chen**, PhD, is a Professor of the China University of Mining and Technology. He is the Associate Dean of Artificial Intelligence Research Institute, China University of Mining and Technology. He is also the Founding Director of the Institute of Bioinformatics, China University of Mining and Technology and Big Data Research Center, China University of Mining and Technology. His research interests include complex disease-related non-coding RNA biomarker prediction, computational models for drug discovery and early detection of human complex disease based on big data and artificial intelligence algorithms.
