# Supplementary material for: Circular RNAs and complex diseases: from experimental results to computational models
Source: Brief Bioinform. 2021 Jul 30;22(6):bbab286. doi: 10.1093/bib/bbab286 (PMC8575014; doi:10.1093/bib/bbab286)
Supplement: Supplementary_materials_bbab286 [file supplementary_materials_bbab286.docx]

**Supplementary materials**

**Databases recording circRNA-disease associations**

***Circ2Traits***

(http://gyanxet-beta.com/circdb/)

Circ2Traits is a database of circRNA-disease associations [1]. The potential associations between circRNAs and diseases are inferred based on disease-related single nucleotide polymorphism (SNP) in circRNA loci and interactions between circRNAs and disease-related miRNAs, respectively. There are 1,951 circRNAs potentially related with 105 diseases in this database.

***Circ2Disease***

(http://bioinformatics.zju.edu.cn/Circ2Disease/index.html)

Circ2Disease is a manually curated database, which records experimentally validated circRNA-human disease associations [2]. Circ2Disease database includes 273 associations between 54 human diseases and 237 circRNAs. For each circRNA-disease association, the database provides the information of disease name, circRNA name, experimental method for detecting the circRNA, a short functional introduction of the relationship, as well as other detailed information.

***CircR2Disease***

(http://bioinfo.snnu.edu.cn/CircR2Disease/)

CircR2Disease is a manually curated database, which contains 725 experimentally supported associations between 100 diseases and 661 circRNAs [3]. The curated information for each circRNA-disease association includes circRNA name, genomic coordinate of circRNA, gene symbol of the overlapping gene of circRNA, disease name, expression patterns of circRNA under the disease state (upregulated or downregulated) and experimental techniques for detecting the circRNA.

***CircRNADisease***

(http://cgga.org.cn:9091/circRNADisease/)

CircRNADisease is a freely available database, which collects experimentally validated circRNA-disease associations from more than 800 published literature [4]. There are a total of 354 associations between 330 circRNAs and 48 diseases, of which 54% are cancers and 27% are cardiovascular and cerebrovascular diseases.

***Circad***

(http://clingen.igib.res.in/circad/)

The database of Circad reports experimentally confirmed associations between circRNAs and diseases [5]. There are 1,388 circRNA-disease associations manually collected from experimental literature involving 150 diseases and 1,388 circRNAs transcribed from 720 genes. The information of each circRNA contains name, genome locus, associated disease and description of experimental method for detecting circRNA.

**Databases providing annotation resources for circRNAs**

***circBase***

(http://www.circbase.org/)

circBase [6] is a freely accessible database colleting numerous circRNAs from studies of large-scale circRNA detection [7-11]. There are 92,375 circRNAs, which are identified from *Caenorhabditis elegans*, *Mus musculus*, *Latimeri* and *Homo sapiens* samples through computational or experimental methods. Users can query the information of circRNAs, such as the genomic position, gene symbols, evidence for the occurrence, and download related data from the web server of circBase.

***CircNet***

(http://circnet.mbc.nctu.edu.tw/)

CircNet is a database of circRNAs identified from transcriptome sequencing data of 464 RNA-seq samples [12]. This database contains 34,000 high-confidence circRNAs detected by using the algorithm proposed by Memczak *et al* [13]. Besides, CircNet can provide the information of circRNA expression profiles, circRNA-miRNA sponge regulatory network, circRNA-gene-miRNA regulatory network and so on.

***deepBase v2.0***

(http://biocenter.sysu.edu.cn/deepBase/)

deepBase v2.0 is an updated database focusing on the small RNAs, lncRNAs and circRNAs identified from deep-sequencing data [14]. The database of deepBase v2.0 annotates 14,867 human circRNAs and provides comprehensive expression and evolution profiles of circRNAs. Researchers could obtain considerable resources of expression and evolution profiles of circRNAs from deepBase v2.0 to uncover circRNA functions.

***circRNADb***

(http://reprod.njmu.edu.cn/circrnadb)

circRNADb is a comprehensive database for human circRNAs [15]. It contains 32,914 circRNAs gathered from diversified resources. The rich information about protein-coding potential of these circRNAs is provided by circRNADb, including predicted internal ribosome entry site (IRES) and potential open reading frame (ORF). In addition, circRNADb also provides the information of circRNAs about genomic information, genome sequence, exon splicing and so on.

***Tissue-Specific CircRNA Database* (*TSCD*)**

(http://gb.whu.edu.cn/TSCD)

TSCD is a public database characterizing the features of tissue-specific (TS) circRNAs [16]. There are 302,853 TS circRNAs identified by three different algorithms, circRNA finder [17], CIRI [18] and find circ [8], in the human and mouse genome. TSCD provides the genomic location and conservation of TS circRNAs, and reports the predicted circRNA-miRNA interactions as well as RNA binding protein sites.

***Cancer-specific circRNA databased* (*CSCD*)**

(http://gb.whu.edu.cn/CSCD)

CSCD is a public database [19] containing 272,152 cancer-specific circRNAs, which are predicted by utilizing four popular algorithms, namely Circexplorer [20], circRNA finder [17], find circ [8] and CIRI2 [21], on RNA-seq data from both cancer and normal cell lines. Researches could find the function and regulation of cancer-associated circRNAs through further exploration.

***CIRCpedia v2***

(https://www.picb.ac.cn/rnomics/circpedia/)

CIRCpedia v2 [22] is an updated comprehensive database including comprehensive annotation for 262,782 circRNAs, which are predicted by the tools of CIRC2 and MapSplice [23] based on 180 RNA-seq datasets across six species. Researchers can download the table of their interested circRNAs listed with the information of CIRCpedia ID, gene, species, location, strand, isoform, expression value, cell line, sequencing type, conservation as well as enrichment fold change, from the web server of CIRCpedia v2.

***exoRBase***

(http://www.exorbase.org/)

exoRBase is a database focusing on circRNAs, mRNAs and lncRNAs in human blood exosomes [24]. The database contains 58,330 circRNAs which are identified by the detection tools of find circ [8] and ACFS [25]. The basic information of circRNAs provided by exoRBase database include circRNA expression profile, expression rank, gene symbol, spliced length and so on.

***CircFunBase***

(http://bis.zju.edu.cn/CircFunBase/)

CircFunBase is a web-accessible database that provides functional annotation for 7,059 manually curated circRNAs, mainly from *Homo sapiens* and *Mus musculus* [26]. The database of CircFunBase can provide the information of circRNA function (including circRNA related diseases and biological regulations), GO annotations, name, expression pattern, position, gene symbol and circRNA-associated miRNAs.

***TRCirc***

(http://www.licpathway.net/TRCirc)

The public database of TRCirc mainly offers the transcription regulation information of circRNAs [27]. The current version of TRCirc contains more than 765,000 transcription factor-circRNA relationships involving 92,375 circRANs and 161 transcription factors. In additions, TRCirc also provides the information of circRNA methylation, circRNA expression and super-enhancers associated with circRNAs.

***circbank***

(http://www.circbank.cn/)

circbank is a publicly available database for human circRNAs, which includes 140,790 human annotated circRNAs collected from circbase website [28]. In the database of circbank, circRNAs are named through a new naming system based on the host genes of circRNAs. In addition, circbank collects other information of circRNAs, namely the conservation of circRNAs, predicted miRNA binding site, mutation of circRNAs, m^6^A modification of circRNAs as well as protein-coding potential of circRNAs.

***CircRNAs in cancer cell lines* (*CircRiC*)**

(https://hanlab.uth.edu/cRic/)

CircRiC is a public database, which focuses on the circRNAs in about 1,000 cancer cell lines across 22 cancer lineages [29]. Specifically, a total of 92,589 circRNAs are detected through utilizing four computational algorithms, namely circRNA finder [17], find circ [8], CIRI2 [21] and CircExplorer2 [30]. Based on these circRNAs, CircRiC provides four interactive modules including integrative analysis, drug response, biogenesis, and expression landscape in its web server, which would facilitate the study of the function of circRNAs for researchers.

***MiOncoCirc***

(https://nguyenjoshvo.github.io/)

MiOncoCirc is an extensive clinical cancer-centric database of circRNAs, providing a novel and comprehensive resource including circRNAs from metastases, primary tumors, and very rare cancer types [31]. A total of 227,056 circRNAs are identified from 2,093 clinical human cancer samples by the detection tool of CIRCexplorer [20].

***VirusCircBase***

(http://www.computationalbiology.cn/ViruscircBase/home.html)

VirusCircBase is the first freely available database of virus circRNAs [32]. VirusCircBase contains 11,924 viral circRNAs, which are identified by the methods of circRNA finder [17], find circ [8] and CIRI2 [21]. Each circRNA entry includes the information of the location, genes involved in the viral circRNA, the abundance, the detection method and so on. VirusCircBase could provide support for researchers to further study virus circRNAs.

1. Ghosal S, Das S, Sen R et al. Circ2Traits: a comprehensive database for circular RNA potentially associated with disease and traits, Front Genet 2013;4.

2. Yao D, Zhang L, Zheng M et al. Circ2Disease: a manually curated database of experimentally validated circRNAs in human disease, Sci Rep 2018;8:11018.

3. Fan C, Lei X, Fang Z et al. CircR2Disease: a manually curated database for experimentally supported circular RNAs associated with various diseases, Database (Oxford) 2018;2018:bay044.

4. Zhao Z, Wang K, Wu F et al. circRNA disease: a manually curated database of experimentally supported circRNA-disease associations, Cell Death Dis 2018;9:475.

5. Rophina M, Sharma D, Poojary M et al. Circad: a comprehensive manually curated resource of circular RNA associated with diseases, Database (Oxford) 2020;2020:baaa019.

6. Glažar P, Papavasileiou P, Rajewsky N. circBase: a database for circular RNAs, Rna 2014;20:1666-1670.

7. Jeck WR, Sorrentino JA, Wang K et al. Circular RNAs are abundant, conserved, and associated with ALU repeats, Rna 2013;19:141-157.

8. Memczak S, Jens M, Elefsinioti A et al. Circular RNAs are a large class of animal RNAs with regulatory potency, Nature 2013;495:333-338.

9. Nitsche A, Doose G, Tafer H et al. Atypical RNAs in the coelacanth transcriptome, J Exp Zool B Mol Dev Evol 2014;322:342-351.

10. Salzman J, Chen RE, Olsen MN et al. Cell-type specific features of circular RNA expression, PLoS Genet 2013;9:e1003777.

11. Zhang Y, Zhang XO, Chen T et al. Circular intronic long noncoding RNAs, Mol Cell 2013;51:792-806.

12. Liu YC, Li JR, Sun CH et al. CircNet: a database of circular RNAs derived from transcriptome sequencing data, Nucleic Acids Res 2016;44:D209-215.

13. Hansen TB, Jensen TI, Clausen BH et al. Natural RNA circles function as efficient microRNA sponges, Nature 2013;495:384-388.

14. Zheng LL, Li JH, Wu J et al. deepBase v2.0: identification, expression, evolution and function of small RNAs, LncRNAs and circular RNAs from deep-sequencing data, Nucleic Acids Res 2016;44:D196-202.

15. Chen X, Han P, Zhou T et al. circRNADb: A comprehensive database for human circular RNAs with protein-coding annotations, Sci Rep 2016;6:34985.

16. Xia S, Feng J, Lei L et al. Comprehensive characterization of tissue-specific circular RNAs in the human and mouse genomes, Brief Bioinform 2017;18:984-992.

17. Westholm JO, Miura P, Olson S et al. Genome-wide analysis of drosophila circular RNAs reveals their structural and sequence properties and age-dependent neural accumulation, Cell Rep 2014;9:1966-1980.

18. Gao Y, Wang J, Zheng Y et al. Comprehensive identification of internal structure and alternative splicing events in circular RNAs, Nat Commun 2016;7:12060.

19. Xia S, Feng J, Chen K et al. CSCD: a database for cancer-specific circular RNAs, Nucleic Acids Res 2018;46:D925-d929.

20. Zhang XO, Dong R, Zhang Y et al. Diverse alternative back-splicing and alternative splicing landscape of circular RNAs, Genome Res 2016;26:1277-1287.

21. Gao Y, Zhang J, Zhao F. Circular RNA identification based on multiple seed matching, Brief Bioinform 2018;19:803-810.

22. Dong R, Ma XK, Li GW et al. CIRCpedia v2: An Updated Database for Comprehensive Circular RNA Annotation and Expression Comparison, Genomics Proteomics Bioinformatics 2018;16:226-233.

23. Wang K, Singh D, Zeng Z et al. MapSplice: accurate mapping of RNA-seq reads for splice junction discovery, Nucleic Acids Res 2010;38:e178.

24. Li S, Li Y, Chen B et al. exoRBase: a database of circRNA, lncRNA and mRNA in human blood exosomes, Nucleic Acids Res 2018;46:D106-d112.

25. You X, Conrad TO. Acfs: accurate circRNA identification and quantification from RNA-Seq data, Sci Rep 2016;6:38820.

26. Meng X, Hu D, Zhang P et al. CircFunBase: a database for functional circular RNAs, Database (Oxford) 2019;2019:baz003.

27. Tang Z, Li X, Zhao J et al. TRCirc: a resource for transcriptional regulation information of circRNAs, Brief Bioinform 2019;20:2327-2333.

28. Liu M, Wang Q, Shen J et al. Circbank: a comprehensive database for circRNA with standard nomenclature, RNA Biol 2019;16:899-905.

29. Ruan H, Xiang Y, Ko J et al. Comprehensive characterization of circular RNAs in ~ 1000 human cancer cell lines, Genome Med 2019;11:55.

30. Zhang XO, Wang HB, Zhang Y et al. Complementary sequence-mediated exon circularization, Cell 2014;159:134-147.

31. Vo JN, Cieslik M, Zhang Y et al. The Landscape of Circular RNA in Cancer, Cell 2019;176:869-881.e813.

32. Cai Z, Fan Y, Zhang Z et al. VirusCircBase: a database of virus circular RNAs, Brief Bioinform 2021;22:2182-2190.
